# Variations of generalized weak contractions in partially ordered *b*-metric space

**DOI:** 10.1186/s13104-022-06208-8

**Published:** 2022-12-03

**Authors:** N. Seshagiri Rao, K. Kalyani

**Affiliations:** Department of Mathematics, School of Applied Sciences & Humanities, Vignan’s Foundation for Science, Technology & Research, Vadlamudi, Andhra Pradesh 522213 India

**Keywords:** Ordered *b*-metric space, Compatible mappings, Mixed *f*-monotone, Fixed points, Primary: 47H10, Secondary: 54H25

## Abstract

**Objectives:**

This paper explored the fixed point results for the mappings satisfying generalized weak contractive conditions in a complete partially ordered *b*-metric space. These contractions are some variations of the work done by the authors (Mituku et al. in BMC Res Notes 13:537. 10.1186/s13104-020-05354-1, 2020; Seshagiri et al. in BMC Res Notes 13:451. 10.1186/s13104-020-05273-1, 2020, BMC Res Notes 14:390. 10.1186/s13104-021-05801-7, 2021, BMC Res Notes 14:263. 10.1186/s13104-021-05649-x, 2021) in the same context. To validate the results a few examples are provided.

**Result:**

The aim of this work is to prove some fixed point results of the self mappings in ordered *b*-metric space satisfying variant generalized weak contraction conditions. These results generalize some known results in the provided literature.

## Introduction

A *b*-metric space or a metric type space is one of the most influential generalizations of the usual metric space. It was first initiated by Bakhtin [11] in 1989. Later, this concept has been used extensively by Czerwik [16] in his work and also generalized the Banach contraction principle in a complete *b*-metric space. Thereafter many researchers improved and generalized the fixed point results for single and multi-valued operators in ordered *b*-metric space by considering necessary topological properties, the readers may refer the works from [1, 7, 8, 10, 20–23, 34] and the references therein.

In ordered metric space, Bhaskar et al. [13] have been introduced first the concept of coupled fixed points for certain mappings and applied their results to boundary value problems for obtaining the unique solutions. Lakshmikantham et al. [24] have been initiated the concept of coupled coincidence and coupled common fixed point results for nonlinear contractive mappings with monotone property in partially ordered complete metric space, which generalized and extended the results of [13]. Later, there has been a lot of generalizations and extensions for the results of coupled fixed points and coupled coincidence points in various ordered spaces, some of such works can be found from the articles [4–6, 9, 14, 15, 17–19, 25–27]. Recently, some results on fixed point, coincidence point and coupled coincidence points for the mappings satisfying generalized weak contraction contractions in the context of partially ordered *b*-metric space with topological properties have been investigated by Belay Mituku et al. [12], Seshagiri Rao et al. [28, 32, 33]. In [2] Aftab Alam et al. have generalized some frequently used metrical notations such as completeness, continuity, compatibility etc. to order-theoretic settings especially in ordered metric spaces besides introduced some new notions such as the ICC, DCC, MCC properties etc. and utilized these relatively weaker notions to prove some coincidence theorems for Byod-Wong type contractions. More extended results on coincidence point have been investigated by Aftab Alam et al. [3] in ordered metric spaces whereas neither the whole space nor the range subspaces are required to be complete. Instead they used the completeness of a subspace of ordered metric space satisfying suitable conditions.

In the present work, we proved some fixed point results for the self mappings satisfying a generalized weak contractive condition in a complete partially ordered *b*-metric space. The obtained results generalized and extended the results of [12, 28, 32, 33] and some existing results in the literature. A few examples are illustrated to support findings.

## Mathematical preliminaries

The following definitions and results are frequently used in the upcoming study.

### **Definition 1**

[29, 30] An operator $d{:} \ {\mathscr {U}} \times {\mathscr {U}}\!\! \rightarrow [0, +\infty )$, where ${\mathscr {U}}\!\!$ is a non-empty set is said to be a *b*-metric, if it satisfies the properties given below

- (i) $$d({\mathcalligra{a}}_{\,1},\  {\mathcalligra{a}}_{\,2})=0$$ $$\iff $$ $$ {\mathcalligra{a}}_{\,1}= {\mathcalligra{a}}_{\,2}$$,
- (ii) $$d({{\mathcalligra{a}}_{\,1}},\ {\mathcalligra{a}}_{\,2})=d({{\mathcalligra{a}}_{\,2}},\  {\mathcalligra{a}}_{\,1})$$,
- (iii) $d({\mathcalligra{a}}_{\,1},\ {\mathcalligra{a}}_{\,2}) \le  {\mathcalligra{s}} \left[ d({\mathcalligra{a}}_{\,1},\; {\mathcalligra{a}}_{\,3})+d({\mathcalligra{a}}_{\,3},\; {\mathcalligra{a}}_{\,2})\right]$, for all ${\mathcalligra{a}}_{\,1},\; {\mathcalligra{a}}_{\,2},\; {\mathcalligra{a}}_{\,3}\in {\mathscr {U}}\!\!$ and for some real number ${\mathcalligra{s}} \ge 1$.

Then $({\mathscr {U}}\!\!,d,\; {\mathcalligra{s}})$ is known as a *b*-metric space. Further, $({\mathscr {U}}\!\!,d,\; {\mathcalligra{s}}, \preceq )$ is a partially ordered *b*-metric space if $({\mathscr {U}}\!\!,\preceq )$ is a partially ordered set.

### **Definition 2**

[31] Let $({\mathscr {U}}\!\!,d,\; {\mathcalligra{s}})$ be a *b*-metric space. Then

1. a sequence $\{\; {\mathcalligra{a}}_{\,n}\}$ is said to converge to ${\mathcalligra{a}}$, if $\lim \limits _{n \rightarrow +\infty }d({\mathcalligra{a}}_{\,n},\; {\mathcalligra{a}})=0$ and written as $\lim \limits _{n \rightarrow +\infty }\; {\mathcalligra{a}}_{\,n}= {\mathcalligra{a}}$.
2. $\{\; {\mathcalligra{a}}_{\,n}\}$ is said to be a Cauchy sequence in ${\mathscr {U}}\!\!$, if $\lim \limits _{n,m \rightarrow +\infty }d({\mathcalligra{a}}_{\,n},\; {\mathcalligra{a}}_{\,m})=0$.
3. $({\mathscr {U}}\!\!,d,\; {\mathcalligra{s}})$ is said to be complete, if every Cauchy sequence in it is convergent.

### **Definition 3**

[33] If the metric *d* is complete then $({\mathscr {U}}\!\!,d,\; {\mathcalligra{s}},\preceq )$ is called complete partially ordered *b*-metric space (CPO*b*-MS).

### **Definition 4**

[33] Let $({\mathscr {U}}\!\!,\preceq )$ be a partially ordered set and let ${\mathcalligra{f}}\  ,{\mathscr {T}}\!\!:{\mathscr {U}}\!\! \rightarrow {\mathscr {U}}\!\!$ are two mappings. Then

1. ${\mathscr {T}}\!\!$ is called a monotone non-decreasing, if ${\mathscr {T}}\!\!({\mathcalligra{a}})\preceq {\mathscr {T}}\!\!({\mathcalligra{b}}\,)$ for all ${\mathcalligra{a}},\; {\mathcalligra{b}} \, \in {\mathscr {U}}\!\!$ with ${\mathcalligra{a}} \preceq \; {\mathcalligra{b}}$.
2. an element ${\mathcalligra{a}} \in {\mathscr {U}}\!\!$ is called a coincidence (common fixed) point of ${\mathscr {T}}\!\!$ and $$ {\mathcalligra{f}}\  $$, if ${\mathcalligra{f}}\  {\mathcalligra{a}}={\mathscr {T}}\!\!\; {\mathcalligra{a}}\,({\mathcalligra{f}}\; {\mathcalligra{a}}={\mathscr {T}}\!\!\; {\mathcalligra{a}}= {\mathcalligra{a}})$.
3. $$ {\mathcalligra{f}}\  $$ and ${\mathscr {T}}\!\!$ are called commuting, if ${\mathcalligra{f}}\  {\mathscr {T}}\!\!\; {\mathcalligra{a}}={\mathscr {T}}\!\!\; {\mathcalligra{f}}\   {\mathcalligra{a}}$, for all ${\mathcalligra{a}}\in {\mathscr {U}}\!\!$.
4. $$ {\mathcalligra{f}}\  $$ and ${\mathscr {T}}\!\!$ are called compatible, if any sequence $\{\; {\mathcalligra{a}}_{\,n}\}$ with $\lim \nolimits _{n \rightarrow +\infty }\; {\mathcalligra{f}}\   {\mathcalligra{a}}_{\,n}= \lim \nolimits _{n \rightarrow +\infty }{\mathscr {T}}\!\!\; {\mathcalligra{a}}_{\,n} =\mu ,\, \text {for}\, \mu \in {\mathscr {U}}\!\!$ then $\lim \nolimits _{n \rightarrow +\infty } d({\mathscr {T}}\!\!\; {\mathcalligra{f}}\   {\mathcalligra{a}}_{\,n},\; {\mathcalligra{f}}\  {\mathscr {T}}\!\!\; {\mathcalligra{a}}_{\,n}) =0$.
5. a pair of self maps $({\mathcalligra{f}}\  ,{\mathscr {T}}\!\!)$ is called weakly compatible, if ${\mathcalligra{f}}\ {\mathscr {T}}\!\!\; {\mathcalligra{a}}={\mathscr {T}}\!\! {\mathcalligra{f}}\  {\mathcalligra{a}}$, when ${\mathscr {T}}\!\!\; {\mathcalligra{a}}= {\mathcalligra{f}}\   {\mathcalligra{a}}$ for some ${\mathcalligra{a}} \in {\mathscr {U}}\!\!$.
6. ${\mathscr {T}}\!\!$ is called monotone $$ {\mathcalligra{f}}\  $$-nondecreasing, if
  $$\begin{aligned} \; {\mathcalligra{f}}\   {\mathcalligra{a}} \preceq \; {\mathcalligra{f}}\   {\mathcalligra{b}}\, \implies {\mathscr {T}}\!\!\; {\mathcalligra{a}} \preceq {\mathscr {T}}\!\!\; {\mathcalligra{b}},\,\text {for any} \,\; {\mathcalligra{a}},\; {\mathcalligra{b}} \in {\mathscr {U}}\!\!. \end{aligned}$$
7. a non empty set ${\mathscr {U}}\!\!$ is called a well ordered set, if very two elements of it are comparable i.e., ${\mathcalligra{a}} \preceq \; {\mathcalligra{b}}$ or ${\mathcalligra{b}} \preceq \; {\mathcalligra{a}}$, for all ${\mathcalligra{a}}, \; {\mathcalligra{b}} \in {\mathscr {U}}\!\!$.

### **Definition 5**

[4, 24] Suppose $({\mathscr {U}}\!\!, \preceq )$ be a partially ordered set and let ${\mathscr {T}}\!\!: {\mathscr {U}}\!\! \times {\mathscr {U}}\!\! \rightarrow {\mathscr {U}}\!\!$ and ${\mathcalligra{f}}\  : {\mathscr {U}}\!\! \rightarrow {\mathscr {U}}\!\!$ be two mappings. Then

1. ${\mathscr {T}}\!\!$ has the mixed $$ {\mathcalligra{f}}\  $$-monotone property, if ${\mathscr {T}}\!\!$ is non-decreasing $$ {\mathcalligra{f}}\  $$-monotone in its first argument and is non-increasing $$ {\mathcalligra{f}}\  $$-monotone in its second argument, that is for any ${\mathcalligra{a}}, \; {\mathcalligra{b}} \,\in {\mathscr {U}}\!\!$
  $$\begin{aligned} &\; {\mathcalligra{a}}_{\,1}, \; {\mathcalligra{a}}_{\,2} \in {\mathscr {U}}\!\!,\,\,\; {\mathcalligra{f}}\   {\mathcalligra{a}}_{\,1} \preceq \; {\mathcalligra{f}}\   {\mathcalligra{a}}_{\,2} \implies {\mathscr {T}}\!\!({\mathcalligra{a}}_{\,1},\; {\mathcalligra{b}}\,)\preceq {\mathscr {T}}\!\!({\mathcalligra{a}}_{\,2},\; {\mathcalligra{b}} \,)\, \text {and} \\ {}&\; {\mathcalligra{b}}_{\,1}, \; {\mathcalligra{b}}_{\,2} \in {\mathscr {U}}\!\!,\,\,\; {\mathcalligra{f}}\   {\mathcalligra{b}}_{\,1} \preceq \; {\mathcalligra{f}}\   {\mathcalligra{b}}_{\,2} \implies {\mathscr {T}}\!\!({\mathcalligra{a}},\; {\mathcalligra{b}}_{\,1})\succeq {\mathscr {T}}\!\!({\mathcalligra{a}},\; {\mathcalligra{b}}_{\,2}). \end{aligned}$$
  Suppose, if $$ {\mathcalligra{f}}\  $$ is an identity mapping then ${\mathscr {T}}\!\!$ is said to have the mixed monotone property.
2. an element $({\mathcalligra{a}},\; {\mathcalligra{b}})\, \in {\mathscr {U}}\!\! \times {\mathscr {U}}\!\!$ is called a coupled coincidence point of ${\mathscr {T}}\!\!$ and $$ {\mathcalligra{f}}\  $$, if ${\mathscr {T}}\!\!({\mathcalligra{a}},\; {\mathcalligra{b}})\,= {\mathcalligra{f}}\   {\mathcalligra{a}}$ and ${\mathscr {T}}\!\!({\mathcalligra{b}},\; {\mathcalligra{a}})= {\mathcalligra{f}}\   {\mathcalligra{b}}$. In particular, if $$ {\mathcalligra{f}}\  $$ is an identity mapping then $({\mathcalligra{a}},\; {\mathcalligra{b}}\,)$ is a coupled fixed point of ${\mathscr {T}}\!\!$.
3. element ${\mathcalligra{a}} \in {\mathscr {U}}\!\!$ is called a common fixed point of ${\mathscr {T}}\!\!$ and $$ {\mathcalligra{f}}\  $$, if ${\mathscr {T}}\!\!({\mathcalligra{a}},\; {\mathcalligra{a}})= {\mathcalligra{f}}\   {\mathcalligra{a}}= {\mathcalligra{a}}$.
4. ${\mathscr {T}}\!\!$ and $$ {\mathcalligra{f}}\  $$ are commutative, if for all ${\mathcalligra{a}}, \; {\mathcalligra{b}} \in {\mathscr {U}}\!\!$, ${\mathscr {T}}\!\!({\mathcalligra{f}}\; {\mathcalligra{a}},\; {\mathcalligra{f}}\   {\mathcalligra{b}}\,) = {\mathcalligra{f}}\  ({\mathscr {T}}\!\!\; {\mathcalligra{a}},{\mathscr {T}}\!\!\; {\mathcalligra{b}}\,)$.
5. ${\mathscr {T}}\!\!$ and $$ {\mathcalligra{f}}\  $$ are said to be compatible, if
  $$\begin{aligned} &\lim \limits _{n \rightarrow +\infty } d({\mathcalligra{f}}\,\,({\mathscr {T}}\!\!({\mathcalligra{a}}_{\,n}, \; {\mathcalligra{b}}_{\,n})), {\mathscr {T}}\!\!({\mathcalligra{f}}\; {\mathcalligra{a}}_{\,n},\; {\mathcalligra{f}}\   {\mathcalligra{b}}_{\,n}))=0\,\text {and}\,\\ {}&\lim \limits _{n \rightarrow +\infty } d({\mathcalligra{f}}\,\,({\mathscr {T}}\!\!({\mathcalligra{b}}_{\,n},\; {\mathcalligra{a}}_{\,n})), {\mathscr {T}}\!\!({\mathcalligra{f}}\; {\mathcalligra{b}}_{\,n},\; {\mathcalligra{f}}\   {\mathcalligra{a}}_{\,n}))=0, \end{aligned} $$
  whenever $\{\; {\mathcalligra{a}}_{\,n}\}$ and $\{\; {\mathcalligra{b}}_{\,n}\}$ are any two sequences in ${\mathscr {U}}\!\!$ such that $\lim \nolimits _{n \rightarrow +\infty } {\mathscr {T}}\!\!({\mathcalligra{a}}_{\,n},\; {\mathcalligra{b}}_{\,n})=\lim \nolimits _{n \rightarrow +\infty } \; {\mathcalligra{f}}\   {\mathcalligra{a}}_{\,n}= {\mathcalligra{a}}$ and $\lim \nolimits _{n \rightarrow +\infty } {\mathscr {T}}\!\!({\mathcalligra{b}}_{\,n},\; {\mathcalligra{a}}_{\,n})=\lim \nolimits _{n \rightarrow +\infty } \; {\mathcalligra{f}}\   {\mathcalligra{b}}_{\,n}= {\mathcalligra{b}}$, for any ${\mathcalligra{a}}, \; {\mathcalligra{b}} \,\in {\mathscr {U}}\!\!$.

The following lemma will be used in the case of sequences convergence in a *b*-metric space $({\mathscr {U}}\!\!,d,\; {\mathcalligra{s}},\preceq )$.

### **Lemma 6**

[4]* Let* $({\mathscr {U}}\!\!,d,\; {\mathcalligra{s}},\preceq )$ *be a b-metric space with* $s>1$* and suppose that* $\{\; {\mathcalligra{a}}_{\,n} \}$* and* $\{\; {\mathcalligra{b}}_{\,n}\}$ *are b*-*convergent to* ${\mathcalligra{a}}$* and* ${\mathcalligra{b}}$* respectively. Then*

$$\begin{aligned} \frac{1}{\; {\mathcalligra{s}}^2}d({\mathcalligra{a}},\; {\mathcalligra{b}})\le \lim \limits _{n \rightarrow +\infty } \inf d({\mathcalligra{a}}_{\,n},\; {\mathcalligra{b}}_{\,n}) \le \lim \limits _{n \rightarrow +\infty } \sup d({\mathcalligra{a}}_{\,n},\; {\mathcalligra{b}}_{\,n})\le \; {\mathcalligra{s}}^2 d({\mathcalligra{a}},\; {\mathcalligra{b}}\,). \end{aligned}$$

*In particular, if* ${\mathcalligra{a}}= {\mathcalligra{b}}\,$,* then* $\lim \nolimits _{n \rightarrow +\infty } d({\mathcalligra{a}}_{\,n},\; {\mathcalligra{b}}_{\, n})=0$.* Moreover, for each* $\tau \in {\mathscr {U}}\!\!$,* we have*

$$\begin{aligned} \frac{1}{\; {\mathcalligra{s}}}d({\mathcalligra{a}},\tau )\le \lim \limits _{n \rightarrow +\infty } \inf d({\mathcalligra{a}}_{\,n},\tau ) \le \lim \limits _{n \rightarrow +\infty } \sup d({\mathcalligra{a}}_{\,n},\tau )\le \; {\mathcalligra{s}} d({\mathcalligra{a}},\tau ). \end{aligned}$$

## Main results

Throughout the paper, we use the following distance functions.

A self mapping $\phi$ defined on $[0, +\infty )$ is said to be an altering distance function, if it satisfies the following conditions:

(i) $\phi$ is a continuous and non-decreasing,
(ii) $$\phi (t)=0$$ $$\iff $$ $$t=0$$.

Let us denote the set of all above altering distance functions on $[0, +\infty )$ by $\Phi$.

Similarly, $\Psi$ denote the set of all operators $\psi :[0, +\infty )\rightarrow [0, +\infty )$ satisfying the following conditions:

(i) $\psi$ is lower semi-continuous,
(ii) $$\psi (t)=0$$ $$\iff $$ $$t=0$$.

Let $({\mathscr {U}}\!\!,d,\; {\mathcalligra{s}},\preceq )$ be a partially ordered *b*-metric space with parameter ${\mathcalligra{s}} > 1$ and let ${\mathscr {T}}\!\!:{\mathscr {U}}\!\! \rightarrow {\mathscr {U}}\!\!$ be a mapping. Set

1 $$\begin{aligned} \begin{aligned} {\mathscr {M}}({\mathcalligra{a}},\; {\mathcalligra{b}}\,)&=\max \{\frac{d({\mathcalligra{b}}\,,{\mathscr {T}}\!\!\; {\mathcalligra{b}}\,) \left[ 1+d({\mathcalligra{a}},{\mathscr {T}}\!\!\; {\mathcalligra{a}})\right] }{1+d({\mathcalligra{a}},\; {\mathcalligra{b}}\,)},\frac{d({\mathcalligra{a}},{\mathscr {T}}\!\!\; {\mathcalligra{b}}\,)+ d({\mathcalligra{b}}\,,{\mathscr {T}}\!\!\; {\mathcalligra{a}})}{2\; {\mathcalligra{s}}}, d({\mathcalligra{a}},{\mathscr {T}}\!\!\; {\mathcalligra{a}}),\\ {}&\,\,\,\,\,\,\,\,\,\,\,\,d({\mathcalligra{b}}\,,{\mathscr {T}}\!\!\; {\mathcalligra{b}}\,), d({\mathcalligra{a}},\; {\mathcalligra{b}}\,)\}, \end{aligned} \end{aligned}$$

and

2 $$\begin{aligned} {\mathscr {N}}({\mathcalligra{a}},\; {\mathcalligra{b}}\,)=\max \{\frac{d({\mathcalligra{b}}\,,{\mathscr {T}}\!\!\; {\mathcalligra{b}}\,) \left[ 1+d({\mathcalligra{a}},{\mathscr {T}}\!\!\; {\mathcalligra{a}})\right] }{1+d({\mathcalligra{a}},\; {\mathcalligra{b}}\,)}, d({\mathcalligra{a}},\; {\mathcalligra{b}}\,)\}. \end{aligned}$$

Let $\phi \in \Phi$ and $\psi \in \Psi$. The mapping ${\mathscr {T}}\!\!$ is called an almost generalized $(\phi ,\psi )_s$-contraction mapping if it satisfies the following condition:

3 $$\begin{aligned} \phi (sd({\mathscr {T}}\!\!\; {\mathcalligra{a}},{\mathscr {T}}\!\!\; {\mathcalligra{b}}\,)) \le \phi ({\mathscr {M}}({\mathcalligra{a}},\; {\mathcalligra{b}}\,))-\psi ({\mathscr {N}}({\mathcalligra{a}},\; {\mathcalligra{b}}\,)), \end{aligned}$$

for any ${\mathcalligra{a}},\; {\mathcalligra{b}}\, \in {\mathscr {U}}\!\!$ with ${\mathcalligra{a}}\preceq \; {\mathcalligra{b}}\,$.

Now, we start this paper with the following fixed point result of a mapping satisfying an almost generalized $(\phi ,\psi )_s$-contraction condition in partially ordered *b*-metric space.

### **Theorem 7**

*Suppose that* $({\mathscr {U}}\!\!,d,\; {\mathcalligra{s}},\preceq )$ *be a CPO b-MS with parameter *${\mathcalligra{s}} > 1$.* Let* ${\mathscr {T}}\!\!:{\mathscr {U}}\!\! \rightarrow {\mathscr {U}}\!\!$* be an almost generalized* $(\phi ,\psi )_{\mathcalligra{s}}$-*contractive mapping, and be continuous, non-decreasing mapping with regards to* $\preceq$.* If there exists certain* ${\mathcalligra{a}}_{\,0} \in {\mathscr {U}}\!\!$* with* ${\mathcalligra{a}}_{\,0} \preceq {\mathscr {T}}\!\!\; {\mathcalligra{a}}_{\,0}$,* then* ${\mathscr {T}}\!\!$* has a fixed point in* ${\mathscr {U}}\!\!$.

### *Proof*

If for some ${\mathcalligra{a}}_{\,0} \in {\mathscr {U}}\!\!$ such that ${\mathscr {T}}\!\!\; {\mathcalligra{a}}_{\,0}= {\mathcalligra{a}}_{\,0}$, then the proof is finished. Assume that ${\mathcalligra{a}}_{\,0} \prec {\mathscr {T}}\!\!\; {\mathcalligra{a}}_{\,0}$, then define a sequence $\{\; {\mathcalligra{a}}_{\,n}\} \subset {\mathscr {U}}\!\!$ by ${\mathcalligra{a}}_{\,n+1}={\mathscr {T}}\!\!\; {\mathcalligra{a}}_{\,n}$, for $n\ge 0$. Since ${\mathscr {T}}\!\!$ is non-decreasing, then by induction we obtain that

4 $$\begin{aligned} \; {\mathcalligra{a}}_{\,0} \prec {\mathscr {T}}\!\!\; {\mathcalligra{a}}_{\,0}= {\mathcalligra{a}}_{\,1}\preceq \cdots \preceq \; {\mathcalligra{a}}_{\,n} \preceq {\mathscr {T}}\!\!\; {\mathcalligra{a}}_{\,n}= {\mathcalligra{a}}_{\,n+1}\preceq ...\,. \end{aligned}$$

If for some $n_0\in {\mathbb {N}}$ such that ${\mathcalligra{a}}_{\,n_0}= {\mathcalligra{a}}_{\,n_0+1}$ then from (4), ${\mathcalligra{a}}_{\,n_0}$ is a fixed point of ${\mathscr {T}}\!\!$ and we have nothing to prove. Suppose that ${\mathcalligra{a}}_{\,n} \ne \; {\mathcalligra{a}}_{\,n+1}$, for all $n \ge 1$. Since $\; {\mathcalligra{a}}_{\,n}>\; {\mathcalligra{a}}_{\,n-1}$ for any $n \ge 1$ and then from contraction condition (3), we have

5 $$\begin{aligned} \begin{aligned} \phi (d({\mathcalligra{a}}_{\,n},\; {\mathcalligra{a}}_{\,n+1}))= \phi (d({\mathscr {T}}\!\!\; {\mathcalligra{a}}_{\,n-1},{\mathscr {T}}\!\!\; {\mathcalligra{a}}_{\,n}))&\le \phi ({\mathcalligra{s}}d({\mathscr {T}}\!\!\; {\mathcalligra{a}}_{\,n-1},{\mathscr {T}}\!\!\; {\mathcalligra{a}}_{\,n})) \\ {}&\le \phi ({\mathscr {M}}({\mathcalligra{a}}_{\,n-1},\; {\mathcalligra{a}}_{\,n}))-\psi ({\mathscr {N}}({\mathcalligra{a}}_{\,n-1},\; {\mathcalligra{a}}_{\,n})). \end{aligned} \end{aligned}$$

From (5), we get

6 $$\begin{aligned} d({\mathcalligra{a}}_{\,n},\; {\mathcalligra{a}}_{\,n+1})= d({\mathscr {T}}\!\!\; {\mathcalligra{a}}_{\,n-1},{\mathscr {T}}\!\!\; {\mathcalligra{a}}_{\,n})\le \frac{1}{\; {\mathcalligra{s}}} {\mathscr {M}}({\mathcalligra{a}}_{\,n-1},\; {\mathcalligra{a}}_{\,n}), \end{aligned}$$

where

$$\begin{aligned} \begin{aligned} {\mathscr {M}}({\mathcalligra{a}}_{\,n-1},\; {\mathcalligra{a}}_{\,n})&=\max \{\frac{d({\mathcalligra{a}}_{\,n},{\mathscr {T}}\!\!\; {\mathcalligra{a}}_{\,n}) \left[ 1+d({\mathcalligra{a}}_{\,n-1},{\mathscr {T}}\!\!\; {\mathcalligra{a}}_{\,n-1})\right] }{1+d({\mathcalligra{a}}_{\,n-1},\; {\mathcalligra{a}}_{\,n})}, \frac{d({\mathcalligra{a}}_{\,n-1},{\mathscr {T}}\!\!\; {\mathcalligra{a}}_{\,n})+ d({\mathcalligra{a}}_{\,n},{\mathscr {T}}\!\!\; {\mathcalligra{a}}_{\,n-1})}{2\; {\mathcalligra{s}}},\\ {}&\,\,\,\,\,\,\,\,\,\,\,\, d({\mathcalligra{a}}_{\,n-1},{\mathscr {T}}\!\!\; {\mathcalligra{a}}_{\,n-1}),d({\mathcalligra{a}}_{\,n},{\mathscr {T}}\!\!\; {\mathcalligra{a}}_{\,n}), d({\mathcalligra{a}}_{\,n-1},\; {\mathcalligra{a}}_{\,n})\} \\ {}&= \max \{d({\mathcalligra{a}}_{\,n},\; {\mathcalligra{a}}_{\,n+1}), \frac{d({\mathcalligra{a}}_{\,n-1},\; {\mathcalligra{a}}_{\,n+1})+ d({\mathcalligra{a}}_{\,n},\; {\mathcalligra{a}}_{\,n})}{2\; {\mathcalligra{s}}}, d({\mathcalligra{a}}_{\,n-1},\; {\mathcalligra{a}}_{\,n})\} \\ {}&\le \max \{d({\mathcalligra{a}}_{\,n},\; {\mathcalligra{a}}_{\,n+1}),\frac{d({\mathcalligra{a}}_{\,n-1},\; {\mathcalligra{a}}_{\,n})+ d({\mathcalligra{a}}_{\,n},\; {\mathcalligra{a}}_{\,n+1})}{2}, d({\mathcalligra{a}}_{\,n-1},\; {\mathcalligra{a}}_{\,n})\} \\ {}&\le \max \{d({\mathcalligra{a}}_{\,n},\; {\mathcalligra{a}}_{\,n+1}),d({\mathcalligra{a}}_{\,n-1},\; {\mathcalligra{a}}_{\,n})\}. \end{aligned} \end{aligned}$$

If $\max \{d({\mathcalligra{a}}_{\,n},\; {\mathcalligra{a}}_{\,n+1}),d({\mathcalligra{a}}_{\,n-1}, \; {\mathcalligra{a}}_{\,n})\}= d({\mathcalligra{a}}_{\,n},\; {\mathcalligra{a}}_{\,n+1})$ for some $n \ge 1$, then from (6) follows

$$\begin{aligned} d({\mathcalligra{a}}_{\,n},\; {\mathcalligra{a}}_{\,n+1})\le \frac{1}{\; {\mathcalligra{s}}} d({\mathcalligra{a}}_{\,n},\; {\mathcalligra{a}}_{\,n+1}), \end{aligned}$$

which is a contradiction. This means that $\max \{d({\mathcalligra{a}}_{\,n},\; {\mathcalligra{a}}_{\,n+1}),d({\mathcalligra{a}}_{\,n-1},\; {\mathcalligra{a}}_{\,n})\}= d({\mathcalligra{a}}_{\,n-1},\; {\mathcalligra{a}}_{\,n})$ for $n \ge 1$. Hence, we obtain from (6) that

$$\begin{aligned} d({\mathcalligra{a}}_{\,n},\; {\mathcalligra{a}}_{\,n+1})\le \frac{1}{\; {\mathcalligra{s}}} d({\mathcalligra{a}}_{\,n-1},\; {\mathcalligra{a}}_{\,n}). \end{aligned}$$

Since, $\frac{1}{\; {\mathcalligra{s}}}\in (0,1)$ then the sequence $\{\; {\mathcalligra{a}}_{\,n}\}$ is a Cauchy sequence by [1, 7] But ${\mathscr {U}}\!\!$ is complete, then there exists some $\mu \in {\mathscr {U}}\!\!$ such that ${\mathcalligra{a}}_{\,n} \rightarrow \mu$.

Also from the continuity of ${\mathscr {T}}\!\!$, we have

$$\begin{aligned} {\mathscr {T}}\!\!\mu ={\mathscr {T}}\!\!(\lim \limits _{n\rightarrow +\infty }\; {\mathcalligra{a}}_{\,n})=\lim \limits _{n\rightarrow +\infty }{\mathscr {T}}\!\!\; {\mathcalligra{a}}_{\,n}=\lim \limits _{n\rightarrow +\infty }\; {\mathcalligra{a}}_{\,n+1}=\mu . \end{aligned}$$

Therefore, $\mu$ is a fixed point of ${\mathscr {T}}\!\!$ in ${\mathscr {U}}\!\!$. $\square$

By relaxing the continuity property of a map ${\mathscr {T}}\!\!$ in Theorem 7, we have the following result.

### **Theorem 8**

*In Theorem* 7,* assume that* ${\mathscr {U}}\!\!$* satisfies*

$$\begin{aligned} \text {if a non-decreasing sequence}\, \{\; {\mathcalligra{a}}_{\,n}\} \rightarrow \mu \in {\mathscr {U}}\!\!,\, \text {then}\, \; {\mathcalligra{a}}_{\,n} \preceq \mu ,\,\text {for all}\, n \in {\mathbb {N}},\,\text {i.e.,}\, \mu =\sup \; {\mathcalligra{a}}_{\,n}. \end{aligned}$$

*Then a non-decreasing mapping* ${\mathscr {T}}\!\!$* has a fixed point in* ${\mathscr {U}}\!\!$.

### *Proof*

From Theorem 7, we construct a non-decreasing Cauchy sequence $\{\; {\mathcalligra{a}}_{\,n}\}$ in ${\mathscr {U}}\!\!$ such that ${\mathcalligra{a}}_{\,n} \rightarrow \mu \in {\mathscr {U}}\!\!$. Therefore from the hypotheses, we have ${\mathcalligra{a}}_{\,n} \preceq \mu$ for all $n \in {\mathbb {N}}$, which implies that $\mu =\sup \; {\mathcalligra{a}}_{\,n}$.

Now, we prove that $\mu$ is a fixed point of ${\mathscr {T}}\!\!$, that is ${\mathscr {T}}\!\!\mu =\mu$. Suppose that ${\mathscr {T}}\!\!\mu \ne \mu$. Let

$$\begin{aligned} \begin{aligned} {\mathscr {M}}({\mathcalligra{a}}_{\,n},\mu )&=\max \{\frac{d(\mu ,{\mathscr {T}}\!\!\mu ) \left[ 1+d({\mathcalligra{a}}_{\,n},{\mathscr {T}}\!\!\; {\mathcalligra{a}}_{\,n})\right] }{1+d({\mathcalligra{a}}_{\,n},\mu )},\frac{d({\mathcalligra{a}}_{\,n},{\mathscr {T}}\!\!\mu )+ d(\mu ,{\mathscr {T}}\!\!\; {\mathcalligra{a}}_{\,n})}{2\; {\mathcalligra{s}}}, d({\mathcalligra{a}}_{\,n},{\mathscr {T}}\!\!\; {\mathcalligra{a}}_{\,n}),\\ {}&\,\,\,\,\,\,\,\,\,\,\,\,\,\,\,d(\mu ,{\mathscr {T}}\!\!\mu ), d({\mathcalligra{a}}_{\,n},\mu )\}, \end{aligned} \end{aligned}$$

and

$$\begin{aligned} {\mathscr {N}}({\mathcalligra{a}}_{\,n},\mu )=\max \{\frac{d(\mu ,{\mathscr {T}}\!\!\mu ) \left[ 1+d({\mathcalligra{a}}_{\,n},{\mathscr {T}}\!\!\; {\mathcalligra{a}}_{\,n})\right] }{1+d({\mathcalligra{a}}_{\,n},\mu )}, d({\mathcalligra{a}}_{\,n},\mu )\}. \end{aligned}$$

Letting $n\rightarrow +\infty$ and using $\lim \nolimits _{n\rightarrow +\infty }\; {\mathcalligra{a}}_{\,n}=\mu$, we get

7 $$\begin{aligned} \lim \limits _{n \rightarrow +\infty }{\mathscr {M}}({\mathcalligra{a}}_{\,n}, \mu )= \max \{d(\mu ,{\mathscr {T}}\!\!\mu ),\frac{d(\mu ,{\mathscr {T}}\!\!\mu )}{2\; {\mathcalligra{s}}},0\}=d(\mu ,{\mathscr {T}}\!\!\mu ), \end{aligned}$$

and

8 $$\begin{aligned} \lim \limits _{n \rightarrow +\infty }{\mathscr {N}}({\mathcalligra{a}}_{\,n}, \mu )= \max \{d(\mu ,{\mathscr {T}}\!\!\mu ),0\}=d(\mu ,{\mathscr {T}}\!\!\mu ). \end{aligned}$$

We know that ${\mathcalligra{a}}_{\,n} \preceq \mu$, for all *n* then from the contraction condition (3), we get

9 $$\begin{aligned} \phi (d({\mathcalligra{a}}_{\,n+1}, {\mathscr {T}}\!\!\mu ))=\phi (d({\mathscr {T}}\!\!\; {\mathcalligra{a}}_{\,n}, {\mathscr {T}}\!\!\mu )\le \phi ({\mathcalligra{s}} d({\mathscr {T}}\!\!\; {\mathcalligra{a}}_{\,n}, {\mathscr {T}}\!\!\mu )\le \phi ({\mathscr {M}}({\mathcalligra{a}}_{\,n}, \mu ))-\psi ({\mathscr {N}}({\mathcalligra{a}}_{\,n}, \mu )). \end{aligned}$$

Letting $n \rightarrow +\infty$ and from the equations (7) and (8), we get

10 $$\begin{aligned} \phi (d(\mu ,{\mathscr {T}}\!\!\mu )) \le \phi (d(\mu ,{\mathscr {T}}\!\!\mu ))-\psi (d(\mu ,{\mathscr {T}}\!\!\mu ))< \phi (d(\mu ,{\mathscr {T}}\!\!\mu )), \end{aligned}$$

which is a contradiction under (10). Thus, ${\mathscr {T}}\!\!\mu =\mu$, that is ${\mathscr {T}}\!\!$ has a fixed point $\mu$ in ${\mathscr {U}}\!\!$. $\square$

Now we give the sufficient condition for the uniqueness of the fixed point that exists in Theorems 7 and 8.

11 $$\begin{aligned} \text {every pair of elements has a lower bound or an upper bound.} \end{aligned}$$

This condition is equivalent to,

$$\begin{aligned} \text {for every}\, \; {\mathcalligra{a}}, \; {\mathcalligra{b}}\, \in {\mathscr {U}}\!\!, \,\text {there exists}\, w \in {\mathscr {U}}\!\! \,\text {which is comparable to}\, \; {\mathcalligra{a}}\, \text {and}\, \; {\mathcalligra{b}}\,. \end{aligned}$$

### **Theorem 9**

*In addition to the hypotheses of Theorem* 7* (or Theorem* 8),* condition* (11)* provides the uniqueness of the fixed point of* ${\mathscr {T}}\!\!$* in* ${\mathscr {U}}\!\!$.

### *Proof*

From Theorem 7 (or Theorem 8), we conclude that ${\mathscr {T}}\!\!$ has a nonempty set of fixed points. Suppose that ${\mathcalligra{a}}^{\,*}$ and ${\mathcalligra{b}}^{\,*}$ be two fixed points of ${\mathscr {T}}\!\!$ then, we claim that ${\mathcalligra{a}}^{\,*}= {\mathcalligra{b}}^{\,*}$. Suppose that ${\mathcalligra{a}}^{\,*}\ne \; {\mathcalligra{b}}^{\,*}$, then from the hypothesis we have

12 $$\begin{aligned} \begin{aligned} \phi (d({\mathscr {T}}\!\!\; {\mathcalligra{a}}^{\,*}, {\mathscr {T}}\!\!\; {\mathcalligra{b}}^{\,*}))&\le \phi ({\mathcalligra{s}}d({\mathscr {T}}\!\!\; {\mathcalligra{a}}^{\,*}, {\mathscr {T}}\!\!\; {\mathcalligra{b}}^{\,*})) \le \phi ({\mathscr {M}}({\mathcalligra{a}}^{\,*}, \; {\mathcalligra{b}}^{\,*}))-\psi ({\mathscr {N}}({\mathcalligra{a}}^{\,*}, \; {\mathcalligra{b}}^{\,*})). \end{aligned} \end{aligned}$$

Consequently, we get

13 $$\begin{aligned} d({\mathcalligra{a}}^{\,*}, \; {\mathcalligra{b}}^{\,*})= d({\mathscr {T}}\!\!\; {\mathcalligra{a}}^{\,*}, {\mathscr {T}}\!\!\; {\mathcalligra{b}}^{\,*}) \le \frac{1}{\; {\mathcalligra{s}}} {\mathscr {M}}({\mathcalligra{a}}^{\,*}, \; {\mathcalligra{b}}^{\,*}), \end{aligned}$$

where

$$\begin{aligned} \begin{aligned} {\mathscr {M}}({\mathcalligra{a}}^{\,*},\; {\mathcalligra{b}}^{\,*})&=\max \{\frac{d({\mathcalligra{b}}^{\,*},{\mathscr {T}}\!\!\; {\mathcalligra{b}}^{\,*}) \left[ 1+d({\mathcalligra{a}}^{\,*},{\mathscr {T}}\!\!\; {\mathcalligra{a}}^{\,*})\right] }{1+d({\mathcalligra{a}}^{\,*},\; {\mathcalligra{b}}^{\,*})},\frac{d({\mathcalligra{a}}^{\,*},{\mathscr {T}}\!\!\; {\mathcalligra{b}}^{\,*})+ d({\mathcalligra{b}}^{\,*},{\mathscr {T}}\!\!\; {\mathcalligra{a}}^{\,*})}{2\; {\mathcalligra{s}}}, d({\mathcalligra{a}}^{\,*},{\mathscr {T}}\!\!\; {\mathcalligra{a}}^{\,*}),\\ {}&\,\,\,\,\,\,\,\,\,\,\,\,\,\,d({\mathcalligra{b}}^{\,*},{\mathscr {T}}\!\!\; {\mathcalligra{b}}^{\,*}), d({\mathcalligra{a}}^{\,*},\; {\mathcalligra{b}}^{\,*})\} \\ {}&=\max \{\frac{d({\mathcalligra{b}}^{\,*},\; {\mathcalligra{b}}^{\,*}) \left[ 1+d({\mathcalligra{a}}^{\,*},\; {\mathcalligra{a}}^{\,*})\right] }{1+d({\mathcalligra{a}}^{\,*}, \; {\mathcalligra{b}}^{\,*})},\frac{d({\mathcalligra{a}}^{\,*},\; {\mathcalligra{b}}^{\,*})+ d({\mathcalligra{b}}^{\,*},\; {\mathcalligra{a}}^{\,*})}{2\; {\mathcalligra{s}}}, d({\mathcalligra{a}}^{\,*},\; {\mathcalligra{a}}^{\,*}),\\ {}&\,\,\,\,\,\,\,\,\,\,\,\,\,\,d({\mathcalligra{b}}^{\,*},\; {\mathcalligra{b}}^{\,*}), d({\mathcalligra{a}}^{\,*},\; {\mathcalligra{b}}^{\,*})\} \\ {}&= \max \{0,\frac{d({\mathcalligra{a}}^{\,*},\; {\mathcalligra{b}}^{\,*})}{\; {\mathcalligra{s}}}, d({\mathcalligra{a}}^{\,*},\; {\mathcalligra{b}}^{\,*}) \} \\ {}&=d({\mathcalligra{a}}^{\,*},\; {\mathcalligra{b}}^{\,*}). \end{aligned} \end{aligned}$$

From (13), we obtain that

$$\begin{aligned} d({\mathcalligra{a}}^{\,*}, \; {\mathcalligra{b}}^{\,*}) \le \frac{1}{\; {\mathcalligra{s}}} d({\mathcalligra{a}}^{\,*}, \; {\mathcalligra{b}}^{\,*})<d({\mathcalligra{a}}^{\,*}, \; {\mathcalligra{b}}^{\,*}), \end{aligned}$$

which is a contradiction. Hence, ${\mathcalligra{a}}^{\,*}= \,{\mathcalligra{b}}^{\,*}$. This completes the proof. $\square$

Let $({\mathscr {U}}\!\!,d,\; {\mathcalligra{s}},\preceq )$ be a partially ordered *b*-metric space with parameter ${\mathcalligra{s}} > 1$, and let ${\mathscr {T}}\!\!,\; {\mathcalligra{f}}\  :{\mathscr {U}}\!\! \rightarrow {\mathscr {U}}\!\!$ be two mappings. Set

14 $$\begin{aligned} \begin{aligned} {\mathscr {M}}_{\mathcalligra{f}}\  ({\mathcalligra{a}},\; {\mathcalligra{b}}\,)&=\max \{\frac{d({\mathcalligra{f}}\; {\mathcalligra{b}}\,,{\mathscr {T}}\!\!\; {\mathcalligra{b}}\,) \left[ 1+d({\mathcalligra{f}}\; {\mathcalligra{a}},{\mathscr {T}}\!\!\; {\mathcalligra{a}})\right] }{1+d({\mathcalligra{f}}\; {\mathcalligra{a}},\; {\mathcalligra{f}}\   {\mathcalligra{b}}\,)},\frac{d({\mathcalligra{f}} \; {\mathcalligra{a}},{\mathscr {T}}\!\!\; {\mathcalligra{b}}\,)+ d({\mathcalligra{f}}\; {\mathcalligra{b}}\,,{\mathscr {T}}\!\!\; {\mathcalligra{a}})}{2\; {\mathcalligra{s}}},\\ {}&\,\,\,\,\,\,\,\,\,\,\,\,\,\,\, d({\mathcalligra{f}}\; {\mathcalligra{a}},{\mathscr {T}}\!\!\; {\mathcalligra{a}}),d({\mathcalligra{f}}\; {\mathcalligra{b}}\,,{\mathscr {T}}\!\!\; {\mathcalligra{b}}\,), d({\mathcalligra{f}}\; {\mathcalligra{a}},\; {\mathcalligra{f}}\   {\mathcalligra{b}}\,)\}, \end{aligned} \end{aligned}$$

and

15 $$\begin{aligned} {\mathscr {N}}_{\mathcalligra{f}}\  ({\mathcalligra{a}},\; {\mathcalligra{b}}\,)=\max \{\frac{d({\mathcalligra{f}} \; {\mathcalligra{b}}\,,{\mathscr {T}}\!\!\; {\mathcalligra{b}}\,) \left[ 1+d({\mathcalligra{f}}\; {\mathcalligra{a}},{\mathscr {T}}\!\!\; {\mathcalligra{a}})\right] }{1+d({\mathcalligra{f}}\; {\mathcalligra{a}},\; {\mathcalligra{f}}\   {\mathcalligra{b}}\,)},d({\mathcalligra{f}}\; {\mathcalligra{a}}, \; {\mathcalligra{f}}\   {\mathcalligra{b}}\,)\}. \end{aligned}$$

Now, we introduce the following definition.

### **Definition 10**

*Let* $({\mathscr {U}}\!\!,d,\; {\mathcalligra{s}},\preceq )$ *be a partially ordered b*-*metric space with* ${\mathcalligra{s}} > 1$.* The mapping* ${\mathscr {T}}\!\!:{\mathscr {U}}\!\! \rightarrow {\mathscr {U}}\!\!$* is called a generalized* $(\phi ,\psi )_{\mathcalligra{s}}$-*contraction mapping with respect to* ${\mathcalligra{f}}\  :{\mathscr {U}}\!\! \rightarrow {\mathscr {U}}\!\!$* for some* $\phi \in \Phi$* and* $\psi \in \Psi$,* if*

16 $$\begin{aligned} \phi ({\mathcalligra{s}}d({\mathscr {T}}\!\!\; {\mathcalligra{a}},{\mathscr {T}}\!\!\; {\mathcalligra{b}}\,)) \le \phi ({\mathscr {M}}_{\mathcalligra{f}}\  ({\mathcalligra{a}},\; {\mathcalligra{b}}\,))-\psi ({\mathscr {N}}_{\mathcalligra{f}}\   ({\mathcalligra{a}},\; {\mathcalligra{b}}\,)), \end{aligned}$$

*for any* ${\mathcalligra{a}},\; {\mathcalligra{b}}\, \in {\mathscr {U}}\!\!$* with* ${\mathcalligra{f}}\   {\mathcalligra{a}}\preceq \; {\mathcalligra{f}}\   {\mathcalligra{b}}\,$,* where* ${\mathscr {M}}_{\mathcalligra{f}}\  ({\mathcalligra{a}},\; {\mathcalligra{b}}\,)$* and* ${\mathscr {N}}_{\mathcalligra{f}}\  ({\mathcalligra{a}},\; {\mathcalligra{b}}\,)$* are given by *(14)* and* (15)* respectively*.

### **Theorem 11**

*Suppose that *$({\mathscr {U}}\!\!,d,\; {\mathcalligra{s}},\preceq )$ *be a CPO b*-*MS with* ${\mathcalligra{s}}> 1$.* Let* ${\mathscr {T}}\!\!: {\mathscr {U}}\!\! \rightarrow {\mathscr {U}}\!\!$* be an almost generalized* $(\phi ,\psi )_{\mathcalligra{s}}$-*contractive mapping with respect to* ${\mathcalligra{f}}\  : {\mathscr {U}}\!\! \rightarrow {\mathscr {U}}\!\!$* and*, ${\mathscr {T}}\!\!$* and* $$ {\mathcalligra{f}}\  $$* are continuous such that* ${\mathscr {T}}\!\!$* is a monotone* $$ {\mathcalligra{f}}\  $$-*non decreasing mapping, compatible with* $$ {\mathcalligra{f}}\  $$* and* ${\mathscr {T}}\!\!{\mathscr {U}}\!\! \subseteq \; {\mathcalligra{f}}\  {\mathscr {U}}\!\!$.* If for some* ${\mathcalligra{a}}_{\,0} \in {\mathscr {U}}\!\!$* such that* ${\mathcalligra{f}}\   {\mathcalligra{a}}_{\,0} \preceq {\mathscr {T}}\!\!\; {\mathcalligra{a}}_{\,0}$,* then* ${\mathscr {T}}\!\!$* and* $$ {\mathcalligra{f}}\  $$* have a coincidence point in* ${\mathscr {U}}\!\!$.

### *Proof*

By following the proof of Theorem 2.2 in [9], we construct two sequences $\{\; {\mathcalligra{a}}_{\,n}\}$ and $\{\; {\mathcalligra{b}}_{\,n}\}$ in ${\mathscr {U}}\!\!$ such that

17 $$\begin{aligned} \; {\mathcalligra{b}}_{\,n}={\mathscr {T}}\!\!\; {\mathcalligra{a}}_{\,n}= {\mathcalligra{f}}\   {\mathcalligra{a}}_{\,n+1} \,\text {for all}\,n\ge 0, \end{aligned}$$

for which

18 $$\begin{aligned} \; {\mathcalligra{f}}\   {\mathcalligra{a}}_{\,0} \preceq \; {\mathcalligra{f}}\   {\mathcalligra{a}}_{\,1} \preceq ... \preceq \; {\mathcalligra{f}}\   {\mathcalligra{a}}_{\,n} \preceq \; {\mathcalligra{f}}\   {\mathcalligra{a}}_{\,n+1} \preceq ...\,. \end{aligned}$$

Again from [9], we have to show that

19 $$\begin{aligned} d({\mathcalligra{b}}_{\,n},\; {\mathcalligra{b}}_{\,n+1})\le \lambda d({\mathcalligra{b}}_{\,n-1},\; {\mathcalligra{b}}_{\,n}), \end{aligned}$$

for all $n \ge 1$ and where $\lambda \in [0, \frac{1}{\; {\mathcalligra{s}}})$. Now from (16) and from the equations (17) and (18), we have

20 $$\begin{aligned} \begin{aligned} \phi ({\mathcalligra{s}}d({\mathcalligra{b}}_{\,n},\; {\mathcalligra{b}}_{\,n+1}))&=\phi ({\mathcalligra{s}}d({\mathscr {T}}\!\! \; {\mathcalligra{a}}_{\,n},{\mathscr {T}}\!\!\; {\mathcalligra{a}}_{\,n+1})) \\&\le \phi ({\mathscr {M}}_{\mathcalligra{f}}\  ({\mathcalligra{a}}_{\,n},\; {\mathcalligra{a}}_{\,n+1})) -\psi ({\mathscr {N}}_{\mathcalligra{f}}\  ({\mathcalligra{a}}_{\,n},\; {\mathcalligra{a}}_{\,n+1})), \end{aligned} \end{aligned}$$

where

$$\begin{aligned} \begin{aligned} {\mathscr {M}}_{\mathcalligra{f}}\  ({\mathcalligra{a}}_{\,n},\; {\mathcalligra{a}}_{\,n+1})&=\max \{\frac{d({\mathcalligra{f}}\; {\mathcalligra{a}}_{\,n+1},{\mathscr {T}}\!\!\; {\mathcalligra{a}}_{\,n+1}) \left[ 1+d({\mathcalligra{f}}\; {\mathcalligra{a}}_{\,n},{\mathscr {T}}\!\!\; {\mathcalligra{a}}_{\,n})\right] }{1+d({\mathcalligra{f}}\; {\mathcalligra{a}}_{\,n},\; {\mathcalligra{f}}\   {\mathcalligra{a}}_{\,n+1})},\\&\,\,\,\,\,\,\,\,\,\,\,\,\,\,\,\frac{d({\mathcalligra{f}}\; {\mathcalligra{a}}_{\,n},{\mathscr {T}}\!\!\; {\mathcalligra{a}}_{\,n+1})+ d({\mathcalligra{f}}\; {\mathcalligra{a}}_{\,n+1},{\mathscr {T}}\!\!\; {\mathcalligra{a}}_{\,n})}{2\; {\mathcalligra{s}}}, d({\mathcalligra{f}}\; {\mathcalligra{a}}_{\,n},{\mathscr {T}}\!\!\; {\mathcalligra{a}}_{\,n}),\\&\,\,\,\,\,\,\,\,\,\,\,\,\,\,\,d({\mathcalligra{f}}\; {\mathcalligra{a}}_{\,n+1},{\mathscr {T}}\!\!\; {\mathcalligra{a}}_{\,n+1}), d({\mathcalligra{f}}\; {\mathcalligra{a}}_{\,n},\; {\mathcalligra{f}}\   {\mathcalligra{a}}_{\,n+1})\} \\ {}&=\max \{\frac{d({\mathcalligra{b}}_{\,n},\; {\mathcalligra{b}}_{\,n+1}) \left[ 1+d({\mathcalligra{b}}_{\,n-1}, \; {\mathcalligra{b}}_{\,n})\right] }{1+d({\mathcalligra{b}}_{\,n-1},\; {\mathcalligra{b}}_{\,n})}, \frac{d({\mathcalligra{b}}_{\,n-1},\; {\mathcalligra{b}}_{\,n+1})+d({\mathcalligra{b}}_{\,n},\; {\mathcalligra{b}}_{\,n})}{2\; {\mathcalligra{s}}},d({\mathcalligra{b}}_{\,n-1},\; {\mathcalligra{b}}_{\,n}),\\&\,\,\,\,\,\,\,\,\,\,\,\,d({\mathcalligra{b}}_{\,n},\; {\mathcalligra{b}}_{\,n+1}), d({\mathcalligra{b}}_{\,n-1},\; {\mathcalligra{b}}_{\,n})\} \\ {}&=\max \{d({\mathcalligra{b}}_{\,n},\; {\mathcalligra{b}}_{\,n+1}),\frac{d({\mathcalligra{b}}_{\,n-1},\; {\mathcalligra{b}}_{\,n}) +d({\mathcalligra{b}}_{\,n},\; {\mathcalligra{b}}_{\,n+1})}{2\; {\mathcalligra{s}}},d({\mathcalligra{b}}_{\,n-1},\; {\mathcalligra{b}}_{\,n})\} \\ {}&\le \max \{d({\mathcalligra{b}}_{\,n},\; {\mathcalligra{b}}_{\,n+1}), d({\mathcalligra{b}}_{\,n-1},\; {\mathcalligra{b}}_{\,n})\}, \end{aligned} \end{aligned}$$

and

$$\begin{aligned} \begin{aligned} {\mathscr {N}}_{\mathcalligra{f}}\  ({\mathcalligra{a}}_{\,n},\; {\mathcalligra{a}}_{\,n+1})&=\max \{\frac{d({\mathcalligra{f}} \; {\mathcalligra{a}}_{\,n+1},{\mathscr {T}}\!\!\; {\mathcalligra{a}}_{\,n+1}) \left[ 1+d({\mathcalligra{f}}\; {\mathcalligra{a}}_{\,n},{\mathscr {T}}\!\!\; {\mathcalligra{a}}_{\,n})\right] }{1+d({\mathcalligra{f}} \; {\mathcalligra{a}}_{\,n},\; {\mathcalligra{f}}\   {\mathcalligra{a}}_{\,n+1})},d({\mathcalligra{f}}\; {\mathcalligra{a}}_{\,n},\; {\mathcalligra{f}}\   {\mathcalligra{a}}_{\,n+1})\} \\ {}&=\max \{\frac{d({\mathcalligra{b}}_{\,n},\; {\mathcalligra{b}}_{\,n+1}) \left[ 1+d({\mathcalligra{b}}_{\,n-1}, \; {\mathcalligra{b}}_{\,n})\right] }{1+d({\mathcalligra{b}}_{\,n-1},\; {\mathcalligra{b}}_{\,n})},d({\mathcalligra{b}}_{\,n-1},\; {\mathcalligra{b}}_{\,n})\} \\ {}&=\max \{d({\mathcalligra{b}}_{\,n-1},\; {\mathcalligra{b}}_{\,n}),d({\mathcalligra{b}}_{\,n},\; {\mathcalligra{b}}_{\,n+1})\}. \end{aligned} \end{aligned}$$

Therefore from the equation (20), we get

21 $$\begin{aligned} \phi ({\mathcalligra{s}}d({\mathcalligra{b}}_{\,n},\; {\mathcalligra{b}}_{\,n+1}))\le \phi (\max \{d({\mathcalligra{b}}_{\,n-1}, \; {\mathcalligra{b}}_{\,n}),d({\mathcalligra{b}}_{\,n},\; {\mathcalligra{b}}_{\,n+1})\})-\psi (\max \{d({\mathcalligra{b}}_{\,n-1}, \; {\mathcalligra{b}}_{\,n}),d({\mathcalligra{b}}_{\,n},\; {\mathcalligra{b}}_{\,n+1})\}). \end{aligned}$$

If $0<d({\mathcalligra{b}}_{\,n-1},\; {\mathcalligra{b}}_{\,n})\le d({\mathcalligra{b}}_{\,n},\; {\mathcalligra{b}}_{\,n+1})$ for some $n \in {\mathbb {N}}$, then from (21) we get

22 $$\begin{aligned} \phi ({\mathcalligra{s}}d({\mathcalligra{b}}_{\,n},\; {\mathcalligra{b}}_{\,n+1}))\le \phi (d({\mathcalligra{b}}_{\,n}, \; {\mathcalligra{b}}_{\,n+1}))-\psi (d({\mathcalligra{b}}_{\,n},\; {\mathcalligra{b}}_{\,n+1}))<\phi (d({\mathcalligra{b}}_{\,n},\; {\mathcalligra{b}}_{\,n+1})), \end{aligned}$$

or equivalently

23 $$\begin{aligned} \; {\mathcalligra{s}}d({\mathcalligra{b}}_{\,n},\; {\mathcalligra{b}}_{\,n+1})\le d({\mathcalligra{b}}_{\,n},\; {\mathcalligra{b}}_{\,n+1}), \end{aligned}$$

which is a contradiction. Hence from (21) we have

24 $$\begin{aligned} \; {\mathcalligra{s}}d({\mathcalligra{b}}_{\,n},\; {\mathcalligra{b}}_{\,n+1})\le d({\mathcalligra{b}}_{\,n-1},\; {\mathcalligra{b}}_{\,n}). \end{aligned}$$

Thus equation (19) holds, where $\lambda \in [0,\frac{1}{\; {\mathcalligra{s}}})$. Therefore from (19) and Lemma 3.1 of [21], we conclude that $\{\; {\mathcalligra{b}}_{\,n}\}=\{{\mathscr {T}}\!\!\; {\mathcalligra{a}}_{\,n}\}=\{\; {\mathcalligra{f}}\   {\mathcalligra{a}}_{\,n+1}\}$ is a Cauchy sequence in ${\mathscr {U}}\!\!$ and then converges to some $\mu \in {\mathscr {U}}\!\!$ as ${\mathscr {U}}\!\!$ is complete such that

$$\begin{aligned} \lim \limits _{n \rightarrow +\infty }{\mathscr {T}}\!\!\; {\mathcalligra{a}}_{\,n}=\lim \limits _{n \rightarrow +\infty }\; {\mathcalligra{f}}\   {\mathcalligra{a}}_{\,n+1}=\mu . \end{aligned}$$

Thus by the compatibility of ${\mathscr {T}}\!\!$ and $$ {\mathcalligra{f}}\  $$, we obtain that

25 $$\begin{aligned} \lim \limits _{n \rightarrow +\infty }d({\mathcalligra{f}}({\mathscr {T}}\!\!\; {\mathcalligra{a}}_{\,n}), {\mathscr {T}}\!\!({\mathcalligra{f}}\; {\mathcalligra{a}}_{\,n}))=0, \end{aligned}$$

and from the continuity of ${\mathscr {T}}\!\!$ and $$ {\mathcalligra{f}}\  $$, we have

26 $$\begin{aligned} \lim \limits _{n \rightarrow +\infty 
}\; {\mathcalligra{f}}\  ({\mathscr {T}}\!\!\; {\mathcalligra{a}}_{\,n})= {\mathcalligra{f}}\  \mu ,\,\,\,\,\,\,\,\,\,\,\,\lim \limits _{n \rightarrow +\infty } {\mathscr {T}}\!\!({\mathcalligra{f}}\; {\mathcalligra{a}}_{\,n})={\mathscr {T}}\!\!\mu . \end{aligned}$$

Further by the triangular inequality a metric *d* and from the equations (25) and (26), we get

27 $$\begin{aligned} \frac{1}{\; {\mathcalligra{s}}}d({\mathscr {T}}\!\!\mu ,\; {\mathcalligra{f}}\ mu )\le d({\mathscr {T}}\!\!\mu ,{\mathscr {T}}\!\!({\mathcalligra{f}}\; {\mathcalligra{a}}_{\,n}))+\; {\mathcalligra{s}} d({\mathscr {T}}\!\!({\mathcalligra{f}}\; {\mathcalligra{a}}_{\,n}), \; {\mathcalligra{f}}\ ({\mathscr {T}}\!\!\; {\mathcalligra{a}}_{\,n}))+\; {\mathcalligra{s}}d({\mathcalligra{f}}({\mathscr {T}}\!\!\; {\mathcalligra{a}}_{\,n}), \; {\mathcalligra{f}}\ mu ). \end{aligned}$$

Finally, we arrive at $d({\mathscr {T}}\!\!v,\; {\mathcalligra{f}}\ v)=0$ as $n \rightarrow +\infty$ in (27). Therefore, *v* is a coincidence point of ${\mathscr {T}}\!\!$ and $$ {\mathcalligra{f}}\ $$ in ${\mathscr {U}}\!\!$. $\square$

Relaxing the continuity criteria of $$ {\mathcalligra{f}}\ $$ and ${\mathscr {T}}\!\!$ in Theorem 11, we obtain the following result.

### **Theorem 12**

*In Theorem* 11,* assume that* ${\mathscr {U}}\!\!$* satisfies*

$$\begin{aligned} \begin{aligned} \,\,\,\,\,&\text { for any non-decreasing sequence}\, \{\; {\mathcalligra{f}}\  {\mathcalligra{a}}_{\,n}\}\subset {\mathscr {U}}\!\!\,\text {with}\, \lim \limits _{n \rightarrow +\infty } \; {\mathcalligra{f}}\  {\mathcalligra{a}}_{\,n}= {\mathcalligra{f}}\  {\mathcalligra{a}}\, \text {in}\, \; {\mathcalligra{f}}\ {\mathscr {U}}\!\!, \text {where}\\ {}&\; {\mathcalligra{f}}\ {\mathscr {U}}\!\! \,\text {is a closed subset of}\, {\mathscr {U}}\!\!\,\text {implies that}\, \; {\mathcalligra{f}}\  {\mathcalligra{a}}_{\,n} \preceq \; {\mathcalligra{f}}\  {\mathcalligra{a}}, \; {\mathcalligra{f}}\  {\mathcalligra{a}} \preceq \; {\mathcalligra{f}}\ ({\mathcalligra{f}}\; {\mathcalligra{a}})\,\text {for}\,n \in {\mathbb {N}}. \end{aligned} \end{aligned}$$

*If there exists* ${\mathcalligra{a}}_{\,0} \in {\mathscr {U}}\!\!$* such that* ${\mathcalligra{f}}\  {\mathcalligra{a}}_{\,0} \preceq {\mathscr {T}}\!\!\; {\mathcalligra{a}}_{\,0}$,* then the weakly compatible mappings* ${\mathscr {T}}\!\!$* and* $$ {\mathcalligra{f}}\ $$* have a coincidence point in* ${\mathscr {U}}\!\!$.* Moreover*, ${\mathscr {T}}\!\!$* and* $$ {\mathcalligra{f}}\ $$* have a common fixed point, if* ${\mathscr {T}}\!\!$* and* $$ {\mathcalligra{f}}\ $$* commute at their coincidence points.*

### *Proof*

The sequence, $\{\; {\mathcalligra{b}}_{\,n}\}=\{{\mathscr {T}}\!\!\; {\mathcalligra{a}}_{\,n}\}=\{\; {\mathcalligra{f}}\  {\mathcalligra{a}}_{\,n+1}\}$ is a Cauchy sequence from the proof of Theorem 11. Since ${\mathcalligra{f}}\ {\mathscr {U}}\!\!$ is closed, then there exists some $\mu \in {\mathscr {U}}\!\!$ such that

$$\begin{aligned} \lim \limits _{n \rightarrow +\infty }{\mathscr {T}}\!\!\; {\mathcalligra{a}}_{\,n}=\lim \limits _{n \rightarrow +\infty }\; {\mathcalligra{f}}\  {\mathcalligra{a}}_{\,n+1}= {\mathcalligra{f}}\ mu . \end{aligned}$$

Thus from the hypotheses, we have ${\mathcalligra{f}}\  {\mathcalligra{a}}_{\,n}\preceq \; {\mathcalligra{f}}\ mu$ for all $n \in {\mathbb {N}}$. Now, we have to prove that $\mu$ is a coincidence point of ${\mathscr {T}}\!\!$ and $$ {\mathcalligra{f}}\ $$.

From equation (16), we have

28 $$\begin{aligned} \begin{aligned} \phi ({\mathcalligra{s}}d({\mathscr {T}}\!\!\; {\mathcalligra{a}}_{\,n},{\mathscr {T}}\!\!\mu )) \le \phi ({\mathscr {M}}_{\mathcalligra{f}}\ ({\mathcalligra{a}}_{\,n},\mu ))-\psi ({\mathscr {N}}_{\mathcalligra{f}}\ ({\mathcalligra{a}}_{\,n},\mu )), \end{aligned} \end{aligned}$$

where

$$\begin{aligned} \begin{aligned} {\mathscr {M}}_{\mathcalligra{f}}\ ({\mathcalligra{a}}_{\,n},\mu )&=\max \{\frac{d({\mathcalligra{f}}\,\,\mu ,{\mathscr {T}}\!\!\mu ) \left[ 1+d({\mathcalligra{f}}\; {\mathcalligra{a}}_{\,n},{\mathscr {T}}\!\!\; {\mathcalligra{a}}_{\,n})\right] }{1+d({\mathcalligra{f}}\; {\mathcalligra{a}}_{\,n},\; {\mathcalligra{f}}\,\,\ mu )},\frac{d({\mathcalligra{f}}\; {\mathcalligra{a}}_{\,n},{\mathscr {T}}\!\!\mu )+ d({\mathcalligra{f}}\,\,\mu ,{\mathscr {T}}\!\!\; {\mathcalligra{a}}_{\,n})}{2\; {\mathcalligra{s}}},\\ {}&\,\,\,\,\,\,\,\,\,\,\,\,\,\,\, d({\mathcalligra{f}}\; {\mathcalligra{a}}_{\,n},{\mathscr {T}}\!\!\; {\mathcalligra{a}}_{\,n}),d({\mathcalligra{f}}\,\,\mu ,{\mathscr {T}}\!\!\mu ), d({\mathcalligra{f}}\; {\mathcalligra{a}}_{\,n},\; {\mathcalligra{f}}\ mu )\} \\&\rightarrow \max \{d({\mathcalligra{f}}\,\,\mu ,{\mathscr {T}}\!\!\mu ),\frac{d({\mathcalligra{f}}\,\,\mu ,{\mathscr {T}}\!\!\mu )}{2\; {\mathcalligra{s}}},0,d({\mathcalligra{f}}\,\,\mu ,{\mathscr {T}}\!\!\mu ),0\} \\ {}&= d({\mathcalligra{f}}\,\,\mu ,{\mathscr {T}}\!\!\mu ) \,\text {as}\,n \rightarrow +\infty , \end{aligned} \end{aligned}$$

and

$$\begin{aligned} \begin{aligned} {\mathscr {N}}_{\mathcalligra{f}}\ ({\mathcalligra{a}}_{\,n},\mu )&=\max \{\frac{d({\mathcalligra{f}}\,\,\mu ,{\mathscr {T}}\!\!\mu ) \left[ 1+d({\mathcalligra{f}}\; {\mathcalligra{a}}_{\,n},{\mathscr {T}}\!\!\; {\mathcalligra{a}}_{\,n})\right] }{1+d({\mathcalligra{f}} \; {\mathcalligra{a}}_{\,n},\; {\mathcalligra{f}}\ mu )},d({\mathcalligra{f}}\; {\mathcalligra{a}}_{\,n},\; {\mathcalligra{f}}\ mu )\} \\&\rightarrow \max \{d({\mathcalligra{f}}\,\,\mu ,{\mathscr {T}}\!\!\mu ),0\} \\ {}&= d({\mathcalligra{f}}\,\,\mu ,{\mathscr {T}}\!\!\mu ) \,\text {as}\,n \rightarrow +\infty . \end{aligned} \end{aligned}$$

Therefore the equation (28) becomes

$$\begin{aligned} \phi ({\mathcalligra{s}}\lim \limits _{n \rightarrow +\infty } d({\mathscr {T}}\!\!\; {\mathcalligra{a}}_{\,n},{\mathscr {T}}\!\!\mu ))\le \phi (d({\mathcalligra{f}}\,\,\mu ,{\mathscr {T}}\!\!\mu )) -\psi (d({\mathcalligra{f}}\,\,\mu ,{\mathscr {T}}\!\!\mu ))< \phi (d({\mathcalligra{f}}\,\,\mu ,{\mathscr {T}}\!\!\mu )). \end{aligned}$$

Consequently, we get

29 $$\begin{aligned} \lim \limits _{n \rightarrow +\infty }d({\mathscr {T}}\!\!\; {\mathcalligra{a}}_{\,n},{\mathscr {T}}\!\!\; {\mathcalligra{a}}) < \frac{1}{\; {\mathcalligra{s}}}d({\mathcalligra{f}}\,\,\mu ,{\mathscr {T}}\!\!\mu ). \end{aligned}$$

Further by triangular inequality of a metric *d*, we have

30 $$\begin{aligned} \frac{1}{\; {\mathcalligra{s}}}d({\mathcalligra{f}}\,\,\mu ,{\mathscr {T}}\!\!\mu )\le d({\mathcalligra{f}}\,\,\mu ,{\mathscr {T}}\!\!\; {\mathcalligra{a}}_{\,n})+d({\mathscr {T}}\!\!\; {\mathcalligra{a}}_{\,n},{\mathscr {T}}\!\!\mu ), \end{aligned}$$

thus (29) and (30) lead to contradiction, if ${\mathcalligra{f}}\,\,\mu \ne {\mathscr {T}}\!\!\mu$. Hence, ${\mathcalligra{f}}\,\,\mu ={\mathscr {T}}\!\!\mu$. Let ${\mathcalligra{f}}\ \mu ={\mathscr {T}}\!\!\mu =\rho$, that is ${\mathscr {T}}\!\!$ and $$ {\mathcalligra{f}}\ $$ are commute at $\rho$, then ${\mathscr {T}}\!\!\rho = {\mathscr {T}}\!\!({\mathcalligra{f}}\,\,\mu )= {\mathcalligra{f}}\ ({\mathscr {T}}\!\!\mu )= {\mathcalligra{f}}\ rho$. Since ${\mathcalligra{f}}\ \mu = {\mathcalligra{f}}\ ({\mathcalligra{f}}\,\,\mu )= {\mathcalligra{f}}\ rho$, then by equation (28) with ${\mathcalligra{f}}\ \mu ={\mathscr {T}}\!\!\mu$ and ${\mathcalligra{f}}\ \rho ={\mathscr {T}}\!\!\rho$, we get

$$\begin{aligned} \begin{aligned} \phi ({\mathcalligra{s}}d({\mathscr {T}}\!\!\mu ,{\mathscr {T}}\!\!\rho ))\le \phi ({\mathscr {M}}_{\mathcalligra{f}}\ (\mu ,\rho )) -\psi ({\mathscr {N}}_{\mathcalligra{f}}\ (\mu ,\rho ))<\phi (d({\mathscr {T}}\!\!\mu ,{\mathscr {T}}\!\!\rho )), \end{aligned} \end{aligned}$$

or equivalently,

$$\begin{aligned} \; {\mathcalligra{s}}d({\mathscr {T}}\!\!\mu ,{\mathscr {T}}\!\!\rho ) \le d({\mathscr {T}}\!\!\mu ,{\mathscr {T}}\!\!\rho ), \end{aligned}$$

which is a contradiction, if ${\mathscr {T}}\!\!\mu \ne {\mathscr {T}}\!\!\rho$. Thus, ${\mathscr {T}}\!\!\mu = {\mathscr {T}}\!\!\rho = \rho$. Hence, ${\mathscr {T}}\!\!\mu = \; {\mathcalligra{f}}\ \rho =\rho$, that is $\rho$ is a common fixed point of ${\mathscr {T}}\!\!$ and $$ {\mathcalligra{f}}\ $$. $\square$

### **Definition 13**

Let $({\mathscr {U}}\!\!,d,\; {\mathcalligra{s}},\preceq )$ be a CPO*b*-MS with ${\mathcalligra{s}} > 1$, $\phi \in \Phi$ and $\psi \in \Psi$. A mapping ${\mathscr {T}}\!\!:{\mathscr {U}}\!\! \times {\mathscr {U}}\!\! \rightarrow {\mathscr {U}}\!\!$ is said to be an almost generalized $(\phi ,\psi )_{\mathcalligra{s}}$-contractive mapping with respect to ${\mathcalligra{f}}\ :{\mathscr {U}}\!\! \rightarrow {\mathscr {U}}\!\!$ such that

31 $$\begin{aligned} \phi ({\mathcalligra{s}}^kd({\mathscr {T}}\!\!({\mathcalligra{a}},\; {\mathcalligra{b}}),{\mathscr {T}}\!\! (\rho ,\tau ))\le \phi ({\mathscr {M}}_{\mathcalligra{f}}\ ({\mathcalligra{a}},\; {\mathcalligra{b}},\rho ,\tau )) -\psi ({\mathscr {N}}_{\mathcalligra{f}}\ ({\mathcalligra{a}},\; {\mathcalligra{b}},\rho ,\tau )), \end{aligned}$$

for all ${\mathcalligra{a}},\; {\mathcalligra{b}},\rho ,\tau \in {\mathscr {U}}\!\!$ with ${\mathcalligra{f}}\  {\mathcalligra{a}} \preceq \; {\mathcalligra{f}}\ \rho$ and ${\mathcalligra{f}}\  {\mathcalligra{b}} \succeq \; {\mathcalligra{f}}\ \tau$, $k>2$ where

$$\begin{aligned} \begin{aligned} {\mathscr {M}}_{\mathcalligra{f}}\ ({\mathcalligra{a}},\; {\mathcalligra{b}},\rho ,\tau )&=\max \{\frac{d({\mathcalligra{f}}\,\,\rho ,{\mathscr {T}}\!\!(\rho ,\tau )) \left[ 1+d({\mathcalligra{f}}\; {\mathcalligra{a}},{\mathscr {T}}\!\!({\mathcalligra{a}},\; {\mathcalligra{b}}))\right] }{1+d({\mathcalligra{f}}\; {\mathcalligra{a}},\; {\mathcalligra{f}}\ \rho )},\frac{d({\mathcalligra{f}}\; {\mathcalligra{a}},{\mathscr {T}}\!\!(\rho ,\tau ))+ d({\mathcalligra{f}}\,\,\rho ,{\mathscr {T}}\!\!({\mathcalligra{a}},\; {\mathcalligra{b}}))}{2\; {\mathcalligra{s}}},\\&\,\,\,\,\,\,\,\,\,\,\,\,\,\,\, d({\mathcalligra{f}}\; {\mathcalligra{a}},{\mathscr {T}}\!\!({\mathcalligra{a}},\; {\mathcalligra{b}})), d({\mathcalligra{f}}\,\,\rho ,{\mathscr {T}}\!\!(\rho ,\tau )), d({\mathcalligra{f}}\; {\mathcalligra{a}},\; {\mathcalligra{f}}\ \rho )\}, \end{aligned} \end{aligned}$$

and

$$\begin{aligned} {\mathscr {N}}_{\mathcalligra{f}}\ ({\mathcalligra{a}},\; {\mathcalligra{b}},\rho ,\tau )=\max \{\frac{d({\mathcalligra{f}}\,\,\rho ,{\mathscr {T}}\!\!(\rho ,\tau )) \left[ 1+d({\mathcalligra{f}}\; {\mathcalligra{a}},{\mathscr {T}}\!\!({\mathcalligra{a}},\; {\mathcalligra{b}}))\right] }{1+d({\mathcalligra{f}}\; {\mathcalligra{a}},\; {\mathcalligra{f}}\ \rho )}, d({\mathcalligra{f}}\; {\mathcalligra{a}},\; {\mathcalligra{f}}\ \rho )\}. \end{aligned}$$

### **Theorem 14**

*Let* $({\mathscr {U}}\!\!,d,\; {\mathcalligra{s}},\preceq )$* be a CPOb-MS with* ${\mathcalligra{s}} > 1$.* Suppose that* ${\mathscr {T}}\!\!:{\mathscr {U}}\!\! \times {\mathscr {U}}\!\! \rightarrow {\mathscr {U}}\!\!$* be an almost generalized* $(\phi ,\psi )_{\mathcalligra{s}}$-*contractive mapping with respect to* ${\mathcalligra{f}}\ :{\mathscr {U}}\!\! \rightarrow {\mathscr {U}}\!\!$* and*, ${\mathscr {T}}\!\!$* and* $$ {\mathcalligra{f}}\ $$* are continuous functions such that* ${\mathscr {T}}\!\!$* has the mixed* $$ {\mathcalligra{f}}\ $$-*monotone property and commutes with* $$ {\mathcalligra{f}}\ $$.* Also assume that* ${\mathscr {T}}\!\!({\mathscr {U}}\!\! \times {\mathscr {U}}\!\!) \subseteq \; {\mathcalligra{f}}\ ({\mathscr {U}}\!\!)$.* Then* ${\mathscr {T}}\!\!$* and* $$ {\mathcalligra{f}}\ $$* have a coupled coincidence point in* ${\mathscr {U}}\!\!$,* if there exists* $({\mathcalligra{a}}_{\,0},\; {\mathcalligra{b}}_{\,0}) \in {\mathscr {U}}\!\! \times {\mathscr {U}}\!\!$* such that* ${\mathcalligra{f}}\  {\mathcalligra{a}}_{\,0} \preceq {\mathscr {T}}\!\!({\mathcalligra{a}}_{\,0},\; {\mathcalligra{b}}_{\,0})$* and* ${\mathcalligra{f}}\  {\mathcalligra{b}}_{\,0} \succeq {\mathscr {T}}\!\!({\mathcalligra{b}}_{\,0},\; {\mathcalligra{a}}_{\,0})$.

### *Proof*

From the hypotheses and following the proof of Theorem 2.2 of [9], we construct two sequences $\{\; {\mathcalligra{a}}_{\,n}\}$ and $\{\; {\mathcalligra{b}}_{\,n}\}$ in ${\mathscr {U}}\!\!$ such that

$$\begin{aligned} \; {\mathcalligra{f}}\  {\mathcalligra{a}}_{\,n+1}={\mathscr {T}}\!\!({\mathcalligra{a}}_{\,n},\; {\mathcalligra{b}}_{\,n}), \,\,\,\,\; {\mathcalligra{f}}\  {\mathcalligra{b}}_{\,n+1}={\mathscr {T}}\!\!({\mathcalligra{b}}_{\,n},\; {\mathcalligra{a}}_{\,n}),\,\,\text {for all}\,n\ge 0. \end{aligned}$$

In particular, $\{\; {\mathcalligra{f}}\  {\mathcalligra{a}}_{\,n}\}$ is a non-decreasing and $\{\; {\mathcalligra{f}}\  {\mathcalligra{b}}_{\,n}\}$ is a non-increasing sequences in ${\mathscr {U}}\!\!$. Now from (31) by replacing ${\mathcalligra{a}}= {\mathcalligra{a}}_{\,n}, \; {\mathcalligra{b}}= {\mathcalligra{b}}_{\,n}, \rho = {\mathcalligra{a}}_{\,n+1}, \tau = {\mathcalligra{b}}_{\,n+1}$, we get

32 $$\begin{aligned} \begin{aligned} \phi ({\mathcalligra{s}}^kd({\mathcalligra{f}}\; {\mathcalligra{a}}_{\,n+1},\; {\mathcalligra{f}}\  {\mathcalligra{a}}_{\,n+2}))&=\phi ({\mathcalligra{s}}^kd({\mathscr {T}}\!\!({\mathcalligra{a}}_{\,n},\; {\mathcalligra{b}}_{\,n}), {\mathscr {T}}\!\!({\mathcalligra{a}}_{\,n+1},\; {\mathcalligra{b}}_{\,n+1})))\\ {}&\le \phi ({\mathscr {M}}_{\mathcalligra{f}}\  ({\mathcalligra{a}}_{\,n},\; {\mathcalligra{b}}_{\,n},\; {\mathcalligra{a}}_{\,n+1},\; {\mathcalligra{b}}_{\,n+1}))-\psi ({\mathscr {N}}_{\mathcalligra{f}}\  ({\mathcalligra{a}}_{\,n},\; {\mathcalligra{b}}_{\,n},\; {\mathcalligra{a}}_{\,n+1},\; {\mathcalligra{b}}_{\,n+1})), \end{aligned} \end{aligned}$$

where

$$\begin{aligned} {\mathscr {M}}_{\mathcalligra{f}}\ ({\mathcalligra{a}}_{\,n},\; {\mathcalligra{b}}_{\,n},\; {\mathcalligra{a}}_{\,n+1},\; {\mathcalligra{b}}_{\,n+1})\le \max \{d({\mathcalligra{f}}\; {\mathcalligra{a}}_{\,n},\; {\mathcalligra{f}}\  {\mathcalligra{a}}_{\,n+1}),d({\mathcalligra{f}} \; {\mathcalligra{a}}_{\,n+1},\; {\mathcalligra{f}}\  {\mathcalligra{a}}_{\,n+2})\} \end{aligned}$$

and

$$\begin{aligned} {\mathscr {N}}_{\mathcalligra{f}}\ ({\mathcalligra{a}}_{\,n},\; {\mathcalligra{b}}_{\,n},\; {\mathcalligra{a}}_{\,n+1},\; {\mathcalligra{b}}_{\,n+1})= \max \{d({\mathcalligra{f}}\; {\mathcalligra{a}}_{\,n},\; {\mathcalligra{f}}\  {\mathcalligra{a}}_{\,n+1}), d({\mathcalligra{f}}\; {\mathcalligra{a}}_{\,n+1},\; {\mathcalligra{f}}\  {\mathcalligra{a}}_{\,n+2})\}. \end{aligned}$$

Therefore from (32), we have

33 $$\begin{aligned} \begin{aligned} \phi ({\mathcalligra{s}}^kd({\mathcalligra{f}}\; {\mathcalligra{a}}_{\,n+1},\; {\mathcalligra{f}}\  {\mathcalligra{a}}_{\,n+2}))&\le \phi (\max \{d({\mathcalligra{f}}\; {\mathcalligra{a}}_{\,n},\; {\mathcalligra{f}}\  {\mathcalligra{a}}_{\,n+1}), d({\mathcalligra{f}}\; {\mathcalligra{a}}_{\,n+1},\; {\mathcalligra{f}}\  {\mathcalligra{a}}_{\,n+2})\})\\ {}&\,\,\,\,\,\,\,-\psi (\max \{d({\mathcalligra{f}}\; {\mathcalligra{a}}_{\,n},\; {\mathcalligra{f}}\  {\mathcalligra{a}}_{\,n+1}), d({\mathcalligra{f}}\; {\mathcalligra{a}}_{\,n+1},\; {\mathcalligra{f}}\  {\mathcalligra{a}}_{\,n+2})\}). \end{aligned} \end{aligned}$$

Similarly by taking ${\mathcalligra{a}}= {\mathcalligra{b}}_{\,n+1}, \; {\mathcalligra{b}}= {\mathcalligra{a}}_{\,n+1}, \rho = {\mathcalligra{a}}_{\,n}, \tau = {\mathcalligra{a}}_{\,n}$ in (31), we get

34 $$\begin{aligned} \begin{aligned} \phi ({\mathcalligra{s}}^kd({\mathcalligra{f}}\; {\mathcalligra{b}}_{\,n+1},\; {\mathcalligra{f}}\  {\mathcalligra{b}}_{\,n+2}))&\le \phi (\max \{d({\mathcalligra{f}}\; {\mathcalligra{b}}_{\,n},\; {\mathcalligra{f}}\  {\mathcalligra{b}}_{\,n+1}), d({\mathcalligra{f}}\; {\mathcalligra{b}}_{\,n+1},\; {\mathcalligra{f}}\  {\mathcalligra{b}}_{\,n+2})\})\\ {}&\,\,\,\,\,\,\,-\psi (\max \{d({\mathcalligra{f}}\; {\mathcalligra{b}}_{\,n},\; {\mathcalligra{f}}\  {\mathcalligra{b}}_{\,n+1}), d({\mathcalligra{f}}\; {\mathcalligra{b}}_{\,n+1},\; {\mathcalligra{f}}\  {\mathcalligra{b}}_{\,n+2})\}). \end{aligned} \end{aligned}$$

From the fact that $\max \{\phi (c_{\,1}),\phi (c_2)\}=\phi \{\max \{c_{\,1},c_2\}\}$ for all $c_{\,1},c_2 \in [0,+\infty )$. Then combining (33) and (34), we get

35 $$\begin{aligned} \begin{aligned} \phi ({\mathcalligra{s}}^k \delta _{\,n})&\le \phi (\max \{d({\mathcalligra{f}}\; {\mathcalligra{a}}_{\,n},\; {\mathcalligra{f}}\  {\mathcalligra{a}}_{\,n+1}),d({\mathcalligra{f}} \; {\mathcalligra{a}}_{\,n+1},\; {\mathcalligra{f}}\  {\mathcalligra{a}}_{\,n+2}),d({\mathcalligra{f}}\; {\mathcalligra{b}}_{\,n}, \; {\mathcalligra{f}}\  {\mathcalligra{b}}_{\,n+1}),d({\mathcalligra{f}}\; {\mathcalligra{b}}_{\,n+1},\; {\mathcalligra{f}}\  {\mathcalligra{b}}_{\,n+2})\})\\&-\psi (\max \{d({\mathcalligra{f}}\; {\mathcalligra{a}}_{\,n},\; {\mathcalligra{f}}\  {\mathcalligra{a}}_{\,n+1}),d({\mathcalligra{f}} \; {\mathcalligra{a}}_{\,n+1},\; {\mathcalligra{f}}\  {\mathcalligra{a}}_{\,n+2}),d({\mathcalligra{f}}\; {\mathcalligra{b}}_{\,n}, \; {\mathcalligra{f}}\  {\mathcalligra{b}}_{\,n+1}),d({\mathcalligra{f}}\; {\mathcalligra{b}}_{\,n+1},\; {\mathcalligra{f}}\  {\mathcalligra{b}}_{\,n+2})\}) \end{aligned} \end{aligned}$$

where

36 $$\begin{aligned} \delta _{\,n}=\max \{d({\mathcalligra{f}}\; {\mathcalligra{a}}_{\,n+1},\; {\mathcalligra{f}}\  {\mathcalligra{a}}_{\,n+2}), d({\mathcalligra{f}}\; {\mathcalligra{b}}_{\,n+1},\; {\mathcalligra{f}}\  {\mathcalligra{b}}_{\,n+2})\}. \end{aligned}$$

Let us denote,

37 $$\begin{aligned} \Delta _{\,n}=\max \{d({\mathcalligra{f}}\; {\mathcalligra{a}}_{\,n},\; {\mathcalligra{f}}\  {\mathcalligra{a}}_{\,n+1}), d({\mathcalligra{f}}\; {\mathcalligra{a}}_{\,n+1},\; {\mathcalligra{f}}\  {\mathcalligra{a}}_{\,n+2}),d({\mathcalligra{f}}\; {\mathcalligra{b}}_{\,n}, \; {\mathcalligra{f}}\  {\mathcalligra{b}}_{\,n+1}),d(f\; {\mathcalligra{b}}_{\,n+1},f\; {\mathcalligra{b}}_{\,n+2})\}. \end{aligned}$$

Hence from the equations (33)-(36), we obtain that

38 $$\begin{aligned} \; {\mathcalligra{s}}^k\delta _n\le \Delta _n. \end{aligned}$$

Next, we prove that

39 $$\begin{aligned} \delta _n\le \lambda \delta _{n-1}, \end{aligned}$$

for all $n \ge 1$ and where $\lambda =\frac{1}{\; {\mathcalligra{s}}^k} \in [0,1)$.

Suppose that if $\Delta _n=\delta _n$ then from (38), we get ${\mathcalligra{s}}^k\delta _n\le \delta _n$ which leads to $\delta _n=0$ as ${\mathcalligra{s}}>1$ and hence (39) holds. If $\Delta _n=\max \{d({\mathcalligra{f}}\; {\mathcalligra{a}}_{\,n},\; {\mathcalligra{f}}\  {\mathcalligra{a}}_{\,n+1}), d({\mathcalligra{f}}\; {\mathcalligra{b}}_{\,n},\; {\mathcalligra{f}}\  {\mathcalligra{b}}_{\,n+1})\}$, i.e., $\Delta _n=\delta _{n-1}$ then (38) follows (39).

Now from (38), we obtain that $\delta _n\le \lambda ^n \delta _{\,0}$ and hence,

$$\begin{aligned} d({\mathcalligra{f}}\; {\mathcalligra{a}}_{\,n+1},\; {\mathcalligra{f}}\  {\mathcalligra{a}}_{\,n+2})\le \lambda ^n \delta _{\,0} \,\,\text {and}\,\,d({\mathcalligra{f}}\; {\mathcalligra{b}}_{\,n+1},\; {\mathcalligra{f}}\  {\mathcalligra{b}}_{\,n+2})\le \lambda ^n \delta _{\,0}. \end{aligned}$$

Therefore from Lemma 3.1 of [21], the sequences $\{\; {\mathcalligra{f}}\  {\mathcalligra{a}}_{\,n}\}$ and $\{\; {\mathcalligra{f}}\  {\mathcalligra{b}}_{\,n}\}$ are Cauchy sequences in ${\mathscr {U}}\!\!$. Thus, from Theorem 2.2 of [5], we conclude that ${\mathscr {T}}\!\!$ and $$ {\mathcalligra{f}}\ $$ have a coincidence point in ${\mathscr {U}}\!\!$. $\square$

### **Corollary 15**

*Let* $({\mathscr {U}}\!\!,d,\; {\mathcalligra{s}},\preceq )$* be a CPOb-MS with* ${\mathcalligra{s}} > 1$,* and* ${\mathscr {T}}\!\!:{\mathscr {U}}\!\! \times {\mathscr {U}}\!\! \rightarrow {\mathscr {U}}\!\!$* be a continuous mapping such that* ${\mathscr {T}}\!\!$* has a mixed monotone property. Suppose there exists* $\phi \in \Phi$* and* $\psi \in \Psi$* such that*

$$\begin{aligned} \phi ({\mathcalligra{s}}^kd({\mathscr {T}}\!\!({\mathcalligra{a}},\; {\mathcalligra{b}}),{\mathscr {T}}\!\!(\rho ,\tau ))) \le \phi ({\mathscr {M}}_{\mathcalligra{f}}\ ({\mathcalligra{a}},\; {\mathcalligra{b}},\rho ,\tau )) -\psi ({\mathscr {N}}_{\mathcalligra{f}}\ ({\mathcalligra{a}},\; {\mathcalligra{b}},\rho ,\tau )), \end{aligned}$$

*for all* ${\mathcalligra{a}},\; {\mathcalligra{b}},\rho ,\tau \in {\mathscr {U}}\!\!$* with* ${\mathcalligra{a}} \preceq \rho$* and* ${\mathcalligra{b}} \succeq \tau$, $k>2$* where*

$$\begin{aligned} \begin{aligned} {\mathscr {M}}_{\mathcalligra{f}}\ ({\mathcalligra{a}},\; {\mathcalligra{b}},\rho ,\tau )&=\max \{\frac{d(\rho ,{\mathscr {T}}\!\!(\rho ,\tau )) \left[ 1+d({\mathcalligra{a}},{\mathscr {T}}\!\!({\mathcalligra{a}},\; {\mathcalligra{b}}))\right] }{1+d({\mathcalligra{a}},\rho )},\frac{d({\mathcalligra{a}},{\mathscr {T}}\!\!(\rho ,\tau ))+ d(\rho ,{\mathscr {T}}\!\!({\mathcalligra{a}},\; {\mathcalligra{b}}))}{2\; {\mathcalligra{s}}},\\ {}&\,\,\,\,\,\,\,\,\,\,\,\, d({\mathcalligra{a}},{\mathscr {T}}\!\!({\mathcalligra{a}},\; {\mathcalligra{b}})),d(\rho ,{\mathscr {T}}\!\!(\rho ,\tau )), d({\mathcalligra{a}},\rho )\}, \end{aligned} \end{aligned}$$

*and*

$$\begin{aligned} {\mathscr {N}}_{\mathcalligra{f}}\ ({\mathcalligra{a}},\; {\mathcalligra{b}},\rho ,\tau )=\max \{\frac{d(\rho ,{\mathscr {T}}\!\!(\rho ,\tau )) \left[ 1+d({\mathcalligra{a}},{\mathscr {T}}\!\!({\mathcalligra{a}},\; {\mathcalligra{b}}))\right] }{1+d({\mathcalligra{a}},\rho )}, d({\mathcalligra{a}},\rho )\}. \end{aligned}$$

*Then* ${\mathscr {T}}\!\!$* has a coupled fixed point in* ${\mathscr {U}}\!\!$,* if there exists* $({\mathcalligra{a}}_{\,0},\; {\mathcalligra{b}}_{\,0}) \in {\mathscr {U}}\!\! \times {\mathscr {U}}\!\!$* such that* ${\mathcalligra{a}}_{\,0} \preceq {\mathscr {T}}\!\!({\mathcalligra{a}}_{\,0},\; {\mathcalligra{b}}_{\,0})$* and* ${\mathcalligra{b}}_{\,0} \succeq {\mathscr {T}}\!\!({\mathcalligra{b}}_{\,0},\; {\mathcalligra{a}}_{\,0})$.

### *Proof*

Set ${\mathcalligra{f}}\ =I_{\mathscr {U}}\!\!$ in Theorem  14. $\square$

### **Corollary 16**

*Let* $({\mathscr {U}}\!\!,d,\; {\mathcalligra{s}},\preceq )$* be a CPOb-MS with* ${\mathcalligra{s}} > 1$,* and* ${\mathscr {T}}\!\!:{\mathscr {U}}\!\! \times {\mathscr {U}}\!\! \rightarrow {\mathscr {U}}\!\!$* be a continuous mapping such that* ${\mathscr {T}}\!\!$* has a mixed monotone property. Suppose there exists* $\psi \in \Psi$* such that*

$$\begin{aligned} d({\mathscr {T}}\!\!({\mathcalligra{a}},\; {\mathcalligra{b}}),{\mathscr {T}}\!\!(\rho ,\tau ))\le \frac{1}{\; {\mathcalligra{s}}^k}{\mathscr {M}}_{\mathcalligra{f}}\ ({\mathcalligra{a}},\; {\mathcalligra{b}},\rho ,\tau ) -\frac{1}{\; {\mathcalligra{s}}^k}\psi ({\mathscr {N}}_{\mathcalligra{f}}\ ({\mathcalligra{a}},\; {\mathcalligra{b}},\rho ,\tau )), \end{aligned}$$

*for all* ${\mathcalligra{a}},\; {\mathcalligra{b}},\rho ,\tau \in P$* with* ${\mathcalligra{a}} \preceq \rho$* and* ${\mathcalligra{b}} \succeq \tau$, $k>2$* where*

$$\begin{aligned} \begin{aligned} {\mathscr {M}}_{\mathcalligra{f}}\ ({\mathcalligra{a}},\; {\mathcalligra{b}},\rho ,\tau )&=\max \{\frac{d(\rho ,{\mathscr {T}}\!\!(\rho ,\tau )) \left[ 1+d({\mathcalligra{a}},{\mathscr {T}}\!\!({\mathcalligra{a}},\; {\mathcalligra{b}}))\right] }{1+d({\mathcalligra{a}},\rho )},\frac{d({\mathcalligra{a}},{\mathscr {T}}\!\!(\rho ,\tau ))+ d(\rho ,{\mathscr {T}}\!\!({\mathcalligra{a}},\; {\mathcalligra{b}}))}{2\; {\mathcalligra{s}}},\\ {}&\,\,\,\,\,\,\,\,\,\,\,\, d({\mathcalligra{a}},{\mathscr {T}}\!\!({\mathcalligra{a}},\; {\mathcalligra{b}})),d(\rho ,{\mathscr {T}}\!\!(\rho ,\tau )), d({\mathcalligra{a}},\rho )\}, \end{aligned} \end{aligned}$$

*and*

$$\begin{aligned} {\mathscr {N}}_{\mathcalligra{f}}\ ({\mathcalligra{a}},\; {\mathcalligra{b}},\rho ,\tau )=\max \{\frac{d(\rho ,{\mathscr {T}}\!\!(\rho ,\tau )) \left[ 1+d({\mathcalligra{a}},{\mathscr {T}}\!\!({\mathcalligra{a}},\; {\mathcalligra{b}}))\right] }{1+d({\mathcalligra{a}},\rho )}, d({\mathcalligra{a}},\rho )\}. \end{aligned}$$

*If there exists* $({\mathcalligra{a}}_{\,0},\; {\mathcalligra{b}}_{\,0}) \in {\mathscr {U}}\!\! \times {\mathscr {U}}\!\!$* such that* ${\mathcalligra{a}}_{\,0} \preceq {\mathscr {T}}\!\!({\mathcalligra{a}}_{\,0},\; {\mathcalligra{b}}_{\,0})$* and* ${\mathcalligra{b}}_{\,0} \succeq {\mathscr {T}}\!\!({\mathcalligra{b}}_{\,0},\; {\mathcalligra{a}}_{\,0})$,* then* ${\mathscr {T}}\!\!$* has a coupled fixed point in* ${\mathscr {U}}\!\!$.

### **Theorem 17**

*In addition to Theorem* 14,* if for all *$({\mathcalligra{a}},\; {\mathcalligra{b}}),(r,s) \in {\mathscr {U}}\!\! \times {\mathscr {U}}\!\!$,* there exists *$(c^*,d^*)\in {\mathscr {U}}\!\! \times {\mathscr {U}}\!\!$* such that *$({\mathscr {T}}\!\!(c^*,d^*), {\mathscr {T}}\!\!(d^*,c^*))$* is comparable to* $({\mathscr {T}}\!\!({\mathcalligra{a}},\; {\mathcalligra{b}}), {\mathscr {T}}\!\!({\mathcalligra{b}},\; {\mathcalligra{a}}))$* and to* $({\mathscr {T}}\!\!(r,s),{\mathscr {T}}\!\!(s,r))$,* then* ${\mathscr {T}}\!\!$* and* $$ {\mathcalligra{f}}\ $$* have a unique coupled common fixed point in* ${\mathscr {U}}\!\! \times {\mathscr {U}}\!\!$.

### *Proof*

From Theorem 14, we know that there exists at least one coupled coincidence point in ${\mathscr {U}}\!\!$ for ${\mathscr {T}}\!\!$ and $$ {\mathcalligra{f}}\ $$. Assume that $({\mathcalligra{a}}, \; {\mathcalligra{b}})$ and (*r*, *s*) are two coupled coincidence points of ${\mathscr {T}}\!\!$ and $$ {\mathcalligra{f}}\ $$, i.e., ${\mathscr {T}}\!\!({\mathcalligra{a}}, \; {\mathcalligra{b}})= {\mathcalligra{f}}\  {\mathcalligra{a}}$, ${\mathscr {T}}\!\!({\mathcalligra{b}},\; {\mathcalligra{a}}, )= {\mathcalligra{f}}\  {\mathcalligra{b}}$ and ${\mathscr {T}}\!\!(r,s)= {\mathcalligra{f}}\ r$, ${\mathscr {T}}\!\!(s,r)= {\mathcalligra{f}}\ s$. Now, we have to prove that ${\mathcalligra{f}}\  {\mathcalligra{a}}= {\mathcalligra{f}}\ r$ and ${\mathcalligra{f}}\  {\mathcalligra{b}}= {\mathcalligra{f}}\ s$.

From the hypotheses, there exists $(c^*,d^*)\in {\mathscr {U}}\!\! \times {\mathscr {U}}\!\!$ such that $({\mathscr {T}}\!\!(c^*,d^*), {\mathscr {T}}\!\!(d^*,c^*))$ is comparable to $({\mathscr {T}}\!\!({\mathcalligra{a}},\; {\mathcalligra{b}}), {\mathscr {T}}\!\!({\mathcalligra{b}},\; {\mathcalligra{a}}))$ and to $({\mathscr {T}}\!\!(r,s),{\mathscr {T}}\!\!(s,r))$. Suppose that

$$\begin{aligned} ({\mathscr {T}}\!\!({\mathcalligra{a}},\; {\mathcalligra{b}}), {\mathscr {T}}\!\!({\mathcalligra{b}},\; {\mathcalligra{a}})) \le ({\mathscr {T}}\!\!(c^*,d^*), {\mathscr {T}}\!\!(d^*,c^*)) \,\text {and}\, ({\mathscr {T}}\!\!(r,s),{\mathscr {T}}\!\!(s,r))\le ({\mathscr {T}}\!\!(c^*,d^*), {\mathscr {T}}\!\!(d^*,c^*)). \end{aligned}$$

Let $c^*_{\,0}=c^*$ and $d^*_{\,0}=d^*$ and then choose $(c^*_{\,1},d^*_{\,1}) \in {\mathscr {U}}\!\! \times {\mathscr {U}}\!\!$ as

$$\begin{aligned} \; {\mathcalligra{f}}\ c^*_{\,1}={\mathscr {T}}\!\!(c^*_{\,0},d^*_{\,0}),\,\, \; {\mathcalligra{f}}\ d^*_{\,1}={\mathscr {T}}\!\!(d^*_{\,0},c^*_{\,0})\,\,(n \ge 1). \end{aligned}$$

By repeating the same procedure above, we can obtain two sequences $\{\; {\mathcalligra{f}}\  c^*_{n}\}$ and $\{\; {\mathcalligra{f}}\  d^*_{n}\}$ in ${\mathscr {U}}\!\!$ such that

$$\begin{aligned} \; {\mathcalligra{f}}\ c^*_{n+1}={\mathscr {T}}\!\!(c^*_n,d^*_n),\,\, \; {\mathcalligra{f}}\ d^*_{n+1}={\mathscr {T}}\!\!(d^*_n,c^*_n)\,\,(n \ge 0). \end{aligned}$$

Similarly, define the sequences $\{\; {\mathcalligra{f}}\  {\mathcalligra{a}}_{\,n}\}$, $\{\; {\mathcalligra{f}}\  {\mathcalligra{b}}_{\,n}\}$ and $\{\; {\mathcalligra{f}}\  r_{n}\}$, $\{\; {\mathcalligra{f}}\  s_{n}\}$ as above in ${\mathscr {U}}\!\!$ by setting ${\mathcalligra{a}}_{\,0}= {\mathcalligra{a}}$, ${\mathcalligra{b}}_{\,0}= {\mathcalligra{b}}$ and $r_{\,0}=r$, $s_{\,0}=s$. Further, we have that

$$\begin{aligned} \; {\mathcalligra{f}}\  {\mathcalligra{a}}_{\,n} \rightarrow {\mathscr {T}}\!\!({\mathcalligra{a}},\; {\mathcalligra{b}}),\,\; {\mathcalligra{f}}\  {\mathcalligra{b}}_{\,n} \rightarrow {\mathscr {T}}\!\!({\mathcalligra{b}},\; {\mathcalligra{a}}),\, \; {\mathcalligra{f}}\ r_{n} \rightarrow {\mathscr {T}}\!\!(r,s),\,\; {\mathcalligra{f}}\ s_n \rightarrow {\mathscr {T}}\!\!(s,r)\,\,(n \ge 1). \end{aligned}$$

Since, $({\mathscr {T}}\!\!({\mathcalligra{a}},\; {\mathcalligra{b}}), {\mathscr {T}}\!\!({\mathcalligra{b}},\; {\mathcalligra{a}}))=({\mathcalligra{f}}\; {\mathcalligra{a}},\; {\mathcalligra{f}}\  {\mathcalligra{b}})=({\mathcalligra{f}}\; {\mathcalligra{a}}_{\,1},\; {\mathcalligra{f}}\  {\mathcalligra{b}}_{\,1})$ is comparable to $({\mathscr {T}}\!\!(c^*,d^*), {\mathscr {T}}\!\!(d^*,c^*))=({\mathcalligra{f}}c^*,\; {\mathcalligra{f}}\ d^*)=({\mathcalligra{f}}c^*_{\,1},\; {\mathcalligra{f}}\ d^*_{\,1})$ and hence we get $({\mathcalligra{f}}\; {\mathcalligra{a}}_{\,1},\; {\mathcalligra{f}}\  {\mathcalligra{b}}_{\,1}) \le ({\mathcalligra{f}}c^*_{\,1},\; {\mathcalligra{f}}\ d^*_{\,1})$. Thus, by induction we obtain that

$$\begin{aligned} ({\mathcalligra{f}}\; {\mathcalligra{a}}_{\,n},\; {\mathcalligra{f}}\  {\mathcalligra{b}}_{\,n}) \le ({\mathcalligra{f}}c^*_n,\; {\mathcalligra{f}}\ d^*_n)\,\,(n \ge 0). \end{aligned}$$

Therefore from (31), we have

40 $$\begin{aligned} \begin{aligned} \phi (d({\mathcalligra{f}}\; {\mathcalligra{a}},\; {\mathcalligra{f}}\ c^*_{n+1}))\le \phi ({\mathcalligra{s}}^3d({\mathcalligra{f}}\; {\mathcalligra{a}},\; {\mathcalligra{f}}\ c^*_{n+1}))&= \phi (d({\mathscr {T}}\!\!({\mathcalligra{a}},\; {\mathcalligra{b}}),{\mathscr {T}}\!\!(c^*_n,d^*_n))) \\ {}&\le \phi ({\mathscr {M}}_{\mathcalligra{f}}\ ({\mathcalligra{a}},\; {\mathcalligra{b}},c^*_n,d^*_n)) -\psi ({\mathscr {N}}_{\mathcalligra{f}}\ ({\mathcalligra{a}},\; {\mathcalligra{b}},c^*_n,d^*_n)), \end{aligned} \end{aligned}$$

where

$$\begin{aligned} \begin{aligned} {\mathscr {M}}_{\mathcalligra{f}}\ ({\mathcalligra{a}},\; {\mathcalligra{b}},c^*_n,d^*_n)&=\max \{\frac{d({\mathcalligra{f}}c^*_n,{\mathscr {T}}\!\!(c^*_n,d^*_n)) \left[ 1+d({\mathcalligra{f}}\; {\mathcalligra{a}},{\mathscr {T}}\!\!({\mathcalligra{a}},\; {\mathcalligra{b}}))\right] }{1+d({\mathcalligra{f}}\; {\mathcalligra{a}},\; {\mathcalligra{f}}\ c^*_n)},\\ {}&\,\,\,\,\,\,\,\,\,\,\,\,\,\,\,\frac{d({\mathcalligra{f}} \; {\mathcalligra{a}},{\mathscr {T}}\!\!(c^*_n,d^*_n))+ d({\mathcalligra{f}}c^*_n,{\mathscr {T}}\!\!({\mathcalligra{a}},\; {\mathcalligra{b}}))}{2\; {\mathcalligra{s}}},\\ {}&\,\,\,\,\,\,\,\,\,\,\,\,\,\,\, d({\mathcalligra{f}}\; {\mathcalligra{a}},{\mathscr {T}}\!\!({\mathcalligra{a}},\; {\mathcalligra{b}})),d({\mathcalligra{f}}c^*_n, {\mathscr {T}}\!\!(c^*_n,d^*_n)), d({\mathcalligra{f}}\; {\mathcalligra{a}},\; {\mathcalligra{f}}\ c^*_n)\} \\ {}&= \max \{0,\frac{d({\mathcalligra{f}}\; {\mathcalligra{a}},\; {\mathcalligra{f}}\ c^*_n)}{\; {\mathcalligra{s}}},0,0,d({\mathcalligra{f}} \; {\mathcalligra{a}},\; {\mathcalligra{f}}\ c^*_n)\} \\ {}&=d({\mathcalligra{f}}\; {\mathcalligra{a}},\; {\mathcalligra{f}}\ c^*_n) \end{aligned} \end{aligned}$$

and

$$\begin{aligned} \begin{aligned} {\mathscr {N}}_{\mathcalligra{f}}\ ({\mathcalligra{a}},\; {\mathcalligra{b}},c^*_n,d^*_n)&=\max \{\frac{d({\mathcalligra{f}}c^*_n, {\mathscr {T}}\!\!(c^*_n,d^*_n)) \left[ 1+d({\mathcalligra{f}}\; {\mathcalligra{a}},{\mathscr {T}}\!\!({\mathcalligra{a}},\; {\mathcalligra{b}}))\right] }{1+d({\mathcalligra{f}}\; {\mathcalligra{a}},\; {\mathcalligra{f}}\ c^*_n)}, d({\mathcalligra{f}}\; {\mathcalligra{a}},\; {\mathcalligra{f}}\ c^*_n)\} \\ {}&=d({\mathcalligra{f}}\; {\mathcalligra{a}},\; {\mathcalligra{f}}\ c^*_n). \end{aligned} \end{aligned}$$

Thus from (40),

41 $$\begin{aligned} \phi (d({\mathcalligra{f}}\; {\mathcalligra{a}},\; {\mathcalligra{f}}\ c^*_{n+1}))\le \phi (d({\mathcalligra{f}}\; {\mathcalligra{a}}, \; {\mathcalligra{f}}\ c^*_n))-\psi (d({\mathcalligra{f}}\; {\mathcalligra{a}},\; {\mathcalligra{f}}\ c^*_n)). \end{aligned}$$

As by the similar process, we can prove that

42 $$\begin{aligned} \phi (d({\mathcalligra{f}}\; {\mathcalligra{b}},\; {\mathcalligra{f}}\ d^*_{n+1}))\le \phi (d({\mathcalligra{f}}\; {\mathcalligra{b}}, \; {\mathcalligra{f}}\ d^*_n))-\psi (d({\mathcalligra{f}}\; {\mathcalligra{b}},\; {\mathcalligra{f}}\ d^*_n)). \end{aligned}$$

From (41) and (42), we have

43 $$\begin{aligned} \begin{aligned} \phi (\max \{d({\mathcalligra{f}}\; {\mathcalligra{a}},\; {\mathcalligra{f}}\ c^*_{n+1}),d({\mathcalligra{f}}\; {\mathcalligra{b}},\; {\mathcalligra{f}}\ d^*_{n+1})\})&\le \phi (\max \{d({\mathcalligra{f}}\; {\mathcalligra{a}},\; {\mathcalligra{f}}\ c^*_n),d({\mathcalligra{f}}\; {\mathcalligra{b}},\; {\mathcalligra{f}}\ d^*_n)\})\\&\,\,\,\,\,-\psi (\max \{d({\mathcalligra{f}}\; {\mathcalligra{a}},\; {\mathcalligra{f}}\ c^*_n),d({\mathcalligra{f}}\; {\mathcalligra{b}},\; 
{\mathcalligra{f}}\ d^*_n)\}) \\ {}&<\phi (\max \{d({\mathcalligra{f}}\; {\mathcalligra{a}},\; {\mathcalligra{f}}\ c^*_n),d({\mathcalligra{f}}\; {\mathcalligra{b}}, \; {\mathcalligra{f}}\ d^*_n)\}). \end{aligned} \end{aligned}$$

Hence by the property of $\phi$, we get

$$\begin{aligned} \max \{d({\mathcalligra{f}}\; {\mathcalligra{a}},\; {\mathcalligra{f}}\ c^*_{n+1}),d({\mathcalligra{f}}\; {\mathcalligra{b}},\; {\mathcalligra{f}}\ d^*_{n+1})\} <\max 
\{d({\mathcalligra{f}}\; {\mathcalligra{a}},\; {\mathcalligra{f}}\ c^*_n),d({\mathcalligra{f}}\; {\mathcalligra{b}},\; {\mathcalligra{f}}\ d^*_n)\}, \end{aligned}$$

which shows that $\max \{d({\mathcalligra{f}}\; {\mathcalligra{a}},\; {\mathcalligra{f}}\ c^*_n),d({\mathcalligra{f}}\; {\mathcalligra{b}},\; {\mathcalligra{f}}\ d^*_n)\}$ is a decreasing sequence and by a result there exists $\gamma \ge 0$ such that

$$\begin{aligned} \lim \limits _{n \rightarrow +\infty }\max \{d({\mathcalligra{f}}\; {\mathcalligra{a}},\; {\mathcalligra{f}}\ c^*_n),d({\mathcalligra{f}}\; {\mathcalligra{b}},\; {\mathcalligra{f}}\ d^*_n)\} =\gamma . \end{aligned}$$

From (43) taking upper limit as $n \rightarrow +\infty$, we get

$$\begin{aligned} \phi (\gamma )\le \phi (\gamma )-\psi (\gamma ), \end{aligned}$$

from which we get $\psi (\gamma )=0$, implies that $\gamma =0$. Thus,

$$\begin{aligned} \lim \limits _{n \rightarrow +\infty }\max \{d({\mathcalligra{f}}\; {\mathcalligra{a}},\; {\mathcalligra{f}}\ c^*_n),d({\mathcalligra{f}}\; {\mathcalligra{b}},\; {\mathcalligra{f}}\ d^*_n)\} =0. \end{aligned}$$

Consequently, we get

44 $$\begin{aligned} \lim \limits _{n \rightarrow +\infty }d({\mathcalligra{f}}\; {\mathcalligra{a}},\; {\mathcalligra{f}}\ c^*_n) =0 \, \text {and} \,\lim \limits _{n \rightarrow +\infty }d({\mathcalligra{f}}\; {\mathcalligra{b}},\; {\mathcalligra{f}}\ d^*_n) =0. \end{aligned}$$

By similar argument, we get

45 $$\begin{aligned} \lim \limits _{n \rightarrow +\infty }d({\mathcalligra{f}}r,\; {\mathcalligra{f}}\ c^*_n) =0 \, \text {and} \,\lim \limits _{n \rightarrow +\infty }d({\mathcalligra{f}}s,\; {\mathcalligra{f}}\ d^*_n) =0. \end{aligned}$$

Therefore from (44) and (45), we get ${\mathcalligra{f}}\  {\mathcalligra{a}}= {\mathcalligra{f}}\ r$ and ${\mathcalligra{f}}\  {\mathcalligra{b}}= {\mathcalligra{f}}\ s$. Science ${\mathcalligra{f}}\  {\mathcalligra{a}}={\mathscr {T}}\!\!({\mathcalligra{a}},\; {\mathcalligra{b}})$ and ${\mathcalligra{f}}\  {\mathcalligra{b}}={\mathscr {T}}\!\!({\mathcalligra{b}},\; {\mathcalligra{a}})$, then by the commutativity of ${\mathscr {T}}\!\!$ and $$ {\mathcalligra{f}}\ $$, we have

46 $$\begin{aligned} \; {\mathcalligra{f}}\ ({\mathcalligra{f}}\; {\mathcalligra{a}})= \; {\mathcalligra{f}}\ ({\mathscr {T}}\!\!({\mathcalligra{a}},\; {\mathcalligra{b}}))={\mathscr {T}}\!\!({\mathcalligra{f}}\; {\mathcalligra{a}}, \; {\mathcalligra{f}}\  {\mathcalligra{b}})\, \text {and}\,\; {\mathcalligra{f}}\ ({\mathcalligra{f}}\; {\mathcalligra{b}})= \; {\mathcalligra{f}}\ ({\mathscr {T}}\!\!({\mathcalligra{b}},\; {\mathcalligra{a}}))={\mathscr {T}}\!\!({\mathcalligra{f}}\; {\mathcalligra{b}}, \; {\mathcalligra{f}}\  {\mathcalligra{a}}). \end{aligned}$$

Let ${\mathcalligra{f}}\  {\mathcalligra{a}}=a^*$ and ${\mathcalligra{f}}\  {\mathcalligra{b}}=b^*$ then (46) becomes

47 $$\begin{aligned} \; {\mathcalligra{f}}\ (a^*)= {\mathscr {T}}\!\!(a^*,b^*)\, \text {and}\,\; {\mathcalligra{f}}\ (b^*)= {\mathscr {T}}\!\!(b^*,a^*), \end{aligned}$$

which shows that $(a^*,b^*)$ is a coupled coincidence point of ${\mathscr {T}}\!\!$ and $$ {\mathcalligra{f}}\ $$. It follows that ${\mathcalligra{f}}\ (a^*)= {\mathcalligra{f}}\ r$ and ${\mathcalligra{f}}\ (b^*)= {\mathcalligra{f}}\ s$ that is ${\mathcalligra{f}}\ (a^*)=a^*$ and ${\mathcalligra{f}}\ (b^*)=b^*$. Thus from (47), we get $a^*= {\mathcalligra{f}}\ (a^*)= {\mathscr {T}}\!\!(a^*,b^*)$ and $b^*= {\mathcalligra{f}}\ (b^*)= {\mathscr {T}}\!\!(b^*,a^*)$. Therefore, $(a^*,b^*)$ is a coupled common fixed point of ${\mathscr {T}}\!\!$ and $$ {\mathcalligra{f}}\ $$.

For the uniqueness let $(u^*,v^*)$ be another coupled common fixed point of ${\mathscr {T}}\!\!$ and $$ {\mathcalligra{f}}\ $$, then we have $u^*= {\mathcalligra{f}}\ u^*= {\mathscr {T}}\!\!(u^*,v^*)$ and $v^*= {\mathcalligra{f}}\ v^*= {\mathscr {T}}\!\!(v^*,u^*)$. Since $(u^*,v^*)$ is a coupled common fixed point of ${\mathscr {T}}\!\!$ and $$ {\mathcalligra{f}}\ $$, then we obtain that ${\mathcalligra{f}}\ u^*= {\mathcalligra{f}}\  {\mathcalligra{a}}=a^*$ and ${\mathcalligra{f}}\ v^*= {\mathcalligra{f}}\  {\mathcalligra{b}}=b^*$. Thus, $u^*= {\mathcalligra{f}}\ u^*= {\mathcalligra{f}}\ a^*=a^*$ and $v^*= {\mathcalligra{f}}\ v^*= {\mathcalligra{f}}\ b^*=b^*$. Hence the result. $\square$

### **Theorem 18**

*In addition to the hypotheses of Theorem* 17, if ${\mathcalligra{f}}\  {\mathcalligra{a}}_{\,0}$* and* ${\mathcalligra{f}}\  {\mathcalligra{b}}_{\,0}$* are comparable, then* ${\mathscr {T}}\!\!$* and* $$ {\mathcalligra{f}}\ $$* have a unique common fixed point in* ${\mathscr {U}}\!\!$.

### *Proof*

From Theorem 17, ${\mathscr {T}}\!\!$ and $$ {\mathcalligra{f}}\ $$ have a unique coupled common fixed point $({\mathcalligra{a}},\; {\mathcalligra{b}}) \in {\mathscr {U}}\!\!$. Now, it is enough to prove that ${\mathcalligra{a}}= {\mathcalligra{b}}$. From the hypotheses, we have ${\mathcalligra{f}}\  {\mathcalligra{a}}_{\,0}$ and ${\mathcalligra{f}}\  {\mathcalligra{b}}_{\,0}$ are comparable then we assume that ${\mathcalligra{f}}\  {\mathcalligra{a}}_{\,0} \preceq \; {\mathcalligra{f}}\  {\mathcalligra{b}}_{\,0}$. Hence by induction we get ${\mathcalligra{f}}\  {\mathcalligra{a}}_{\,n} \preceq \; {\mathcalligra{f}}\  {\mathcalligra{b}}_{\,n}$ for all $n \ge 0$, where $\{\; {\mathcalligra{f}}\  {\mathcalligra{a}}_{\,n}\}$ and $\{\; {\mathcalligra{f}}\  {\mathcalligra{b}}_{\,n}\}$ are from Theorem 14.

Now by use of Lemma 6, we get

$$\begin{aligned}  \phi ({\mathcalligra{s}}^{k-2}d({\mathcalligra{a}},\; {\mathcalligra{b}}))&=\phi ({\mathcalligra{s}}^k \frac{1}{\; {\mathcalligra{s}}^2}d({\mathcalligra{a}},\; {\mathcalligra{b}})) \le \lim \limits _{n \rightarrow +\infty } \sup \phi ({\mathcalligra{s}}^k d({\mathcalligra{a}}_{\,n+1}, \; {\mathcalligra{b}}_{\,n+1}))\\&= \lim \limits _{n \rightarrow +\infty }\sup \phi ({\mathcalligra{s}}^k d({\mathscr {T}}\!\!({\mathcalligra{a}}_{\,n}, \; {\mathcalligra{b}}_{\,n}),{\mathscr {T}}\!\!({\mathcalligra{b}}_{\,n},\; {\mathcalligra{a}}_{\,n})))\\&\le \lim \limits _{n \rightarrow +\infty }\sup \phi ({\mathscr {M}}_{\mathcalligra{f}}\ ({\mathcalligra{a}}_{\,n}, \; {\mathcalligra{b}}_{\,n},\; {\mathcalligra{b}}_{\,n},\; {\mathcalligra{a}}_{\,n}))-\lim \limits _{n \rightarrow +\infty }\inf \psi ({\mathscr {N}}_{\mathcalligra{f}}\ ({\mathcalligra{a}}_{\,n}, \; {\mathcalligra{b}}_{\,n},\; {\mathcalligra{b}}_{\,n},\; {\mathcalligra{a}}_{\,n}))\\&\le \phi (d({\mathcalligra{a}},\; {\mathcalligra{b}}))-\lim \limits _{n \rightarrow +\infty }\inf \psi ({\mathscr {N}}_{\mathcalligra{f}}\ ({\mathcalligra{a}}_{\,n}, \; {\mathcalligra{b}}_{\,n},\; {\mathcalligra{b}}_{\,n},\; {\mathcalligra{a}}_{\,n})) \\ {}&<\phi (d({\mathcalligra{a}},\; {\mathcalligra{b}})), \end{aligned} $$

which is a contradiction. Thus, ${\mathcalligra{a}}= {\mathcalligra{b}}$, i.e., ${\mathscr {T}}\!\!$ and $$ {\mathcalligra{f}}\ $$ have a common fixed point in ${\mathscr {U}}\!\!$. $\square$

### **Corollary 19**

*Suppose* $({\mathscr {U}}\!\!,d,\; {\mathcalligra{s}},\preceq )$ *be a CPO b*-*MS with parameter* ${\mathcalligra{s}} > 1$.* Let* ${\mathscr {T}}\!\!:{\mathscr {U}}\!\! \rightarrow {\mathscr {U}}\!\!$ *be a continuous, non-decreasing map with regards to* $\preceq$* such that there exists* ${\mathcalligra{a}}_{\,0} \in {\mathscr {U}}\!\!$* with* ${\mathcalligra{a}}_{\,0} \preceq {\mathscr {T}}\!\!\; {\mathcalligra{a}}_{\,0}$.* Suppose that*

48 $$\begin{aligned} \phi ({\mathcalligra{s}}d({\mathscr {T}}\!\!\; {\mathcalligra{a}},{\mathscr {T}}\!\!\; {\mathcalligra{b}})) \le \phi ({\mathscr {M}}({\mathcalligra{a}},\; {\mathcalligra{b}}))-\psi ({\mathscr {M}}({\mathcalligra{a}},\; {\mathcalligra{b}})), \end{aligned}$$

*where* ${\mathscr {M}}({\mathcalligra{a}},\; {\mathcalligra{b}})$* and the conditions upon* $\phi , \psi$* are same as in Theorem* 7.* Then* ${\mathscr {T}}\!\!$* has a fixed point in* ${\mathscr {U}}\!\!$.

### *Proof*

Set ${\mathscr {N}}({\mathcalligra{a}},\; {\mathcalligra{b}})={\mathscr {M}}({\mathcalligra{a}},\; {\mathcalligra{b}})$ in a contraction condition (3) and apply Theorem 7, we have the required proof. $\square$

### Remark 20

(i). The fixed point and its uniqueness exists for a non-decreasing mapping ${\mathscr {T}}\!\!$ in ${\mathscr {U}}\!\!$ satisfying the contraction condition (48) by following Theorems 8 & 9 under the same hypothesis.
(ii). One can obtains the coincidence point, coupled coincidence point and its uniqueness of the mappings ${\mathscr {T}}\!\!$ and $$ {\mathcalligra{f}}\ $$ in ${\mathscr {U}}\!\!$ by following Theorems 11 & 12 and Theorems 14, 17 & 18 from the contraction condition (48) by taking ${\mathscr {M}}({\mathcalligra{a}},\; {\mathcalligra{b}})$, ${\mathscr {M}}_{\mathcalligra{f}}\ ({\mathcalligra{a}},\; {\mathcalligra{b}})$, ${\mathscr {M}}_{\mathcalligra{f}}\ ({\mathcalligra{a}},\; {\mathcalligra{b}}, \rho , \tau )$ and the conditions upon $\phi , \psi$ are same as above defined.

### **Corollary 21**

*Suppose that *$({\mathscr {U}}\!\!,d,\; {\mathcalligra{s}},\preceq )$* be a CPOb-MS with* ${\mathcalligra{s}} > 1$.* Let* ${\mathscr {T}}\!\!:{\mathscr {U}}\!\! \rightarrow {\mathscr {U}}\!\!$* be a continuous, non-decreasing mapping with regards to* $\preceq$.* If there exists* $k \in [0,1)$* and for any* ${\mathcalligra{a}},\; {\mathcalligra{b}} \in {\mathscr {U}}\!\!$* with* ${\mathcalligra{a}} \preceq \; {\mathcalligra{b}}$* such that*

49 $$\begin{aligned} \begin{aligned} d({\mathscr {T}}\!\!\; {\mathcalligra{a}},{\mathscr {T}}\!\!\; {\mathcalligra{b}})&\le \frac{k}{\; {\mathcalligra{s}}}\max \{\frac{d({\mathcalligra{b}},{\mathscr {T}}\!\!\; {\mathcalligra{b}}) \left[ 1+d({\mathcalligra{a}},{\mathscr {T}}\!\!\; {\mathcalligra{a}})\right] }{1+d({\mathcalligra{a}}, \; {\mathcalligra{b}})},\frac{d({\mathcalligra{a}},{\mathscr {T}}\!\!\; {\mathcalligra{b}})+ d({\mathcalligra{b}},{\mathscr {T}}\!\!\; {\mathcalligra{a}})}{2\; {\mathcalligra{s}}},\\&\,\,\,\,\,\,\,\,\,\,\,\,\,\,\,\, d({\mathcalligra{a}},{\mathscr {T}}\!\!\; {\mathcalligra{a}}),d({\mathcalligra{b}},{\mathscr {T}}\!\!\; {\mathcalligra{b}}), d({\mathcalligra{a}},\; {\mathcalligra{b}})\}. \end{aligned} \end{aligned}$$

*If there exists* ${\mathcalligra{a}}_{\,0} \in {\mathscr {U}}\!\!$* with* ${\mathcalligra{a}}_{\,0} \preceq {\mathscr {T}}\!\!\; {\mathcalligra{a}}_{\,0}$,* then* ${\mathscr {T}}\!\!$* has a fixed point in* ${\mathscr {U}}\!\!$.

### *Proof*

Set $\phi (t)=t$ and $\psi (t)=(1-k)t$, for all $t \in (0, +\infty )$ in Corollary 19. $\square$

### **Note 1**

Following Theorem 8, a fixed point exists for a non-decreasing mapping ${\mathscr {T}}\!\!$ in Corollary 21.

We give the following examples of the results obtained in different cases such as continuity and discontinuity of a metric *d* in a space ${\mathscr {U}}\!\!$.

### Example 22

Define a metric $d:{\mathscr {U}}\!\! \times {\mathscr {U}}\!\! \rightarrow {\mathscr {U}}\!\!$ as below and $\le$ is an usual order on ${\mathscr {U}}\!\!$, where ${\mathscr {U}}\!\!=\{1,2,3,4,5,6\}$

$$\begin{aligned} d({\mathcalligra{a}}, \ {\mathcalligra{b}}) &= d({\mathcalligra{b}},\ {\mathcalligra{a}})=0, \ {\text{if}} \ {\mathcalligra{a}}, \ {\mathcalligra{b}}=1,2,3,4,5,6 \,{\text{and}}  \ {\mathcalligra{a}}= {\mathcalligra{b}},\\  d({\mathcalligra{a}}, \ {\mathcalligra{b}}) &= d({\mathcalligra{b}},\  {\mathcalligra{a}})=3, \ if  \ {\mathcalligra{a}}, \  {\mathcalligra{b}}=1,2,3,4,5 \ {\text{and}} \  {\mathcalligra{a}} \ne  {\mathcalligra{b}}, \\ d({\mathcalligra{a}}, \ {\mathcalligra{b}}) &= d({\mathcalligra{b}},\  {\mathcalligra{a}})=12, \ if \   {\mathcalligra{a}}=1,2,3,4 \ {\text{and}} \  {\mathcalligra{b}}=6,\\ d({\mathcalligra{a}}, \ {\mathcalligra{b}}) &= d({\mathcalligra{b}},\ {\mathcalligra{a}})=20, \ if \  {\mathcalligra{a}}=5 \ {\text{and}} \  {\mathcalligra{b}}=6. \end{aligned}$$

Define a map ${\mathscr {T}}\!\!:{\mathscr {U}}\!\! \rightarrow {\mathscr {U}}\!\!$ by ${\mathscr {T}}\!\!1={\mathscr {T}}\!\!2={\mathscr {T}}\!\!3={\mathscr {T}}\!\!4={\mathscr {T}}\!\!5=1, {\mathscr {T}}\!\!6=2$ and let $\phi (t)=\frac{t}{2}$, $\psi (t)=\frac{t}{4}$ for $t \in [0,+\infty )$. Then ${\mathscr {T}}\!\!$ has a fixed point in ${\mathscr {U}}\!\!$.

### *Proof*

It is obvious that for ${\mathcalligra{s}}=2$, $({\mathscr {U}}\!\!,d,\; {\mathcalligra{s}},\preceq )$ is a CPO*b*-MS. Consider the possible cases for ${\mathcalligra{a}}$, ${\mathcalligra{b}}$ in ${\mathscr {U}}\!\!$:

**Case 1.** Suppose ${\mathcalligra{a}}, \; {\mathcalligra{b}} \in \{1,2,3,4,5\}$, ${\mathcalligra{a}}<\; {\mathcalligra{b}}$ then $d({\mathscr {T}}\!\!\; {\mathcalligra{a}},{\mathscr {T}}\!\!\; {\mathcalligra{b}})=d(1,1)=0$. Hence,

$$\begin{aligned} \phi (2d({\mathscr {T}}\!\!\; {\mathcalligra{a}},{\mathscr {T}}\!\!\; {\mathcalligra{b}}))=0 \le \phi ({\mathscr {M}}({\mathcalligra{a}},\; {\mathcalligra{b}}))-\psi ({\mathscr {M}}({\mathcalligra{a}},\; {\mathcalligra{b}})). \end{aligned}$$

**Case 2.** Suppose that ${\mathcalligra{a}} \in \{1,2,3,4,5\}$ and ${\mathcalligra{b}}=6$, then $d({\mathscr {T}}\!\!\; {\mathcalligra{a}},{\mathscr {T}}\!\!\; {\mathcalligra{b}})=d(1,2)=3$, ${\mathscr {M}}(6,5)=20$ and ${\mathscr {M}}({\mathcalligra{a}},6)=12$, for ${\mathcalligra{a}} \in \{1,2,3,4\}$. Therefore, we have the following inequality,

$$\begin{aligned} \phi (2d({\mathscr {T}}\!\!\; {\mathcalligra{a}},{\mathscr {T}}\!\!\; {\mathcalligra{b}})) \le \frac{{\mathscr {M}}({\mathcalligra{a}},\; {\mathcalligra{b}})}{4} =\phi ({\mathscr {M}}({\mathcalligra{a}},\; {\mathcalligra{b}}))-\psi ({\mathscr {M}}({\mathcalligra{a}},\; {\mathcalligra{b}})). \end{aligned}$$

Thus, condition (48) of Corollary 19 holds. Furthermore, the remaining assumptions in Corollary 19 are fulfilled. Hence, ${\mathscr {T}}\!\!$ has a fixed point in ${\mathscr {U}}\!\!$ as Corollary 19 is appropriate to ${\mathscr {T}}\!\!, \phi , \psi$ and $({\mathscr {U}}\!\!,d,\; {\mathcalligra{s}},\preceq )$. $\square$

### Example 23

A metric $d:{\mathscr {U}}\!\! \times {\mathscr {U}}\!\! \rightarrow {\mathscr {U}}\!\!$, where ${\mathscr {U}}\!\!=\{0, 1, \frac{1}{2},\frac{1}{3},\frac{1}{4},...\frac{1}{n},...\}$ with usual order $\le$ is as follows

$$\begin{aligned} \begin{aligned} d({\mathcalligra{a}},\; {\mathcalligra{b}})= {\left\{ \begin{array}{ll} &{} \,0 \,\,\,\,\,\,\,\,\,\,,\,\, if\, \; {\mathcalligra{a}}= {\mathcalligra{b}} \\ &{} \,1 \,\,\,\,\,\,\,\,\,\,,\,\, if\, \; {\mathcalligra{a}}\ne \; {\mathcalligra{b}} \in \{0,1\} \\ &{} \,|\; {\mathcalligra{a}}-\; {\mathcalligra{b}}|\,\,,\,\, if\, \; {\mathcalligra{a}},\; {\mathcalligra{b}} \in \{0, \frac{1}{2n},\frac{1}{2m}: n \ne m \ge 1\} \\ &{} \,2 \,\,\,\,\,\,\,\,\,\,\,,\,\, otherwise. \end{array}\right. } \end{aligned} \end{aligned}$$

A map ${\mathscr {T}}\!\!: {\mathscr {U}}\!\! \rightarrow {\mathscr {U}}\!\!$ be such that ${\mathscr {T}}\!\!0=0, {\mathscr {T}}\!\!\frac{1}{n}=\frac{1}{12n}$ for all $n\ge 1$ and let $\phi (t)=t$, $\psi (t)=\frac{4t}{5}$ for $t \in [0,+\infty )$. Then, ${\mathscr {T}}\!\!$ has a fixed point in ${\mathscr {U}}\!\!$.

### *Proof*

It is obvious that for ${\mathcalligra{s}}=\frac{12}{5}$, $({\mathscr {U}}\!\!,d,\; {\mathcalligra{s}},\preceq )$ is a CPO*b*-MS and also by definition, *d* is discontinuous *b*-metric space. Now for ${\mathcalligra{a}},\; {\mathcalligra{b}} \in {\mathscr {U}}\!\!$ with ${\mathcalligra{a}}<\; {\mathcalligra{b}}$, we have the following cases:

**Case 1**. If ${\mathcalligra{a}}=0$ and ${\mathcalligra{b}}=\frac{1}{n}$, $n \ge 1$, then $d({\mathscr {T}}\!\!\; {\mathcalligra{a}},{\mathscr {T}}\!\!\; {\mathcalligra{b}})=d(0,\frac{1}{12n})=\frac{1}{12n}$ and ${\mathscr {M}}({\mathcalligra{a}},\; {\mathcalligra{b}})=\frac{1}{n}$ or ${\mathscr {M}}({\mathcalligra{a}},\; {\mathcalligra{b}})= \{1,2\}$. Therefore, we have

$$\begin{aligned} \phi \left( \frac{12}{5}d({\mathscr {T}}\!\!\; {\mathcalligra{a}},{\mathscr {T}}\!\!\; {\mathcalligra{b}})\right) \le \frac{{\mathscr {M}}({\mathcalligra{a}},\; {\mathcalligra{b}})}{5} =\phi ({\mathscr {M}}({\mathcalligra{a}},\; {\mathcalligra{b}}))-\psi ({\mathscr {M}}({\mathcalligra{a}},\; {\mathcalligra{b}})). \end{aligned}$$

**Case 2**. If ${\mathcalligra{a}}=\frac{1}{m}$ and ${\mathcalligra{b}}=\frac{1}{n}$ with $m>n\ge 1$, then

$$\begin{aligned} d({\mathscr {T}}\!\!\; {\mathcalligra{a}},{\mathscr {T}}\!\!\; {\mathcalligra{b}})=d(\frac{1}{12m},\frac{1}{12n}) \,\text {and}\, {\mathscr {M}}({\mathcalligra{a}},\; {\mathcalligra{b}})\ge \frac{1}{n}-\frac{1}{m}\, \text {or}\, {\mathscr {M}}({\mathcalligra{a}},\; {\mathcalligra{b}})=2. \end{aligned}$$

Therefore,

$$\begin{aligned} \phi \left( \frac{12}{5}d({\mathscr {T}}\!\!\; {\mathcalligra{a}},{\mathscr {T}}\!\!\; {\mathcalligra{b}})\right) \le \frac{{\mathscr {M}}({\mathcalligra{a}},\; {\mathcalligra{b}})}{5} =\phi ({\mathscr {M}}({\mathcalligra{a}},\; {\mathcalligra{b}}))-\psi ({\mathscr {M}}({\mathcalligra{a}},\; {\mathcalligra{b}})). \end{aligned}$$

Hence, condition (48) of Corollary 19 and remaining assumptions are satisfied. Thus, ${\mathscr {T}}\!\!$ has a fixed point in ${\mathscr {U}}\!\!$. $\square$

### Example 24

Let ${\mathscr {U}}\!\!=C[a,b]$ be the set of all continuous functions. Let us define a *b*-metric *d* on ${\mathscr {U}}\!\!$ by

$$\begin{aligned} d(\theta _{1},\theta _2)=\sup _{t \in [a,b]}\{|\theta _{1}(t)-\theta _2(t)|^2\} \end{aligned}$$

for all $\theta _{1},\theta _2 \in {\mathscr {U}}\!\!$ with partial order $\preceq$ defined by $\theta _{1} \preceq \theta _2$ if $a\le \theta _{1}(t) \le \theta _2 (t)\le b$, for all $t \in [a,b]$, $0 \le a<b$. Let ${\mathscr {T}}\!\!:{\mathscr {U}}\!\! \rightarrow {\mathscr {U}}\!\!$ be a mapping defined by ${\mathscr {T}}\!\! \theta = \frac{\theta }{5}, \theta \in {\mathscr {U}}\!\!$ and the two altering distance functions by $\phi (t)=t$, $\psi (t)=\frac{t}{3}$, for any $t \in [0, +\infty ]$. Then ${\mathscr {T}}\!\!$ has a unique fixed point in ${\mathscr {U}}\!\!$.

### *Proof*

From the hypotheses, it is clear that $({\mathscr {U}}\!\!,d,\; {\mathcalligra{s}},\preceq )$ is a CPO*b*-MS with parameter ${\mathcalligra{s}}=2$ and fulfill all conditions of Corollary 19 and Remark 20. Furthermore for any $\theta _{1},\theta _2 \in {\mathscr {U}}\!\!$, the function $\min (\theta _{1},\theta _2) (t)=\min \{\theta _{1}(t),\theta _2(t)\}$ is also continuous and the conditions of Corollary 19 and Remark 20 are satisfied. Hence, ${\mathscr {T}}\!\!$ has a unique fixed point $\theta =0$ in ${\mathscr {U}}\!\!$. $\square$

## Limitations

The existence and uniqueness of a fixed point for a self mapping satisfying a generalized weak contraction in CPO*b*-MS is proved. Furthermore, the results are extended for obtaining the coincidence point and coupled coincidence point for two mappings in the same context. The results can be further extended

- $\bullet$ to triple and quadruple mappings for fixed points and
- $\bullet$ to discuss the results in various spaces with necessary topological properties.

## Data Availability

Not applicable.
